# Optimizing SUV Analysis: A Multicenter Study on Preclinical FDG-PET/CT Highlights the Impact of Standardization

**DOI:** 10.1007/s11307-024-01927-9

**Published:** 2024-06-21

**Authors:** Claudia Kuntner, Carlos Alcaide, Dimitris Anestis, Jens P. Bankstahl, Herve Boutin, David Brasse, Filipe Elvas, Duncan Forster, Maritina G. Rouchota, Adriana Tavares, Mari Teuter, Thomas Wanek, Lena Zachhuber, Julia G. Mannheim

**Affiliations:** 1 Department of Biomedical Imaging and Image-Guided Therapy, Medical University of Vienna, Waehringer Guertel 18-20, 1090 Vienna, Vienna, Austria; 2 Medical Imaging Cluster (MIC), Medical University of Vienna, Vienna, Austria; 3 University of Edinburgh, Edinburgh, UK; 4 BIOEMTECH, Athens, Greece; 5 Hannover Medical School, Hannover, Germany; 6 Division of Neuroscience & Experimental Psychology, Faculty of Biology, Medicine and Health, University of Manchester, Manchester, UK; 7 INSERM, UMR 1253, iBrainUniversité de Tours, Tours, France; 8 Institut Pluridisciplinaire Hubert Curien, UMR7178, Université de Strasbourg, CNRS, Strasbourg, France; 9 Molecular Imaging Center Antwerp, University of Antwerpen, Antwerp, Belgium; 10 Division of Informatics, Imaging and Data Sciences, Manchester Molecular Imaging Centre, The University of Manchester, Manchester, UK; 11 Department of Preclinical Imaging and Radiopharmacy Werner Siemens Imaging Center, Eberhard-Karls University Tuebingen, Tuebingen, Germany; 12 Cluster of Excellence iFIT (EXC 2180) “Image Guided and Functionally Instructed Tumor Therapies”, Tuebingen, Germany

**Keywords:** Multicenter, Image analysis, Reproducibility, PET/CT, Preclinical imaging

## Abstract

**Purpose:**

Preclinical imaging, with translational potential, lacks a standardized method for defining volumes of interest (VOIs), impacting data reproducibility. The aim of this study was to determine the interobserver variability of VOI sizes and standard uptake values (SUV_mean_ and SUV_max_) of different organs using the same [^18^F]FDG-PET and PET/CT datasets analyzed by multiple observers. In addition, the effect of a standardized analysis approach was evaluated.

**Procedures:**

In total, 12 observers (4 beginners and 8 experts) analyzed identical preclinical [^18^F]FDG-PET-only and PET/CT datasets according to their local default image analysis protocols for multiple organs. Furthermore, a standardized protocol was defined, including detailed information on the respective VOI size and position for multiple organs, and all observers reanalyzed the PET/CT datasets following this protocol.

**Results:**

Without standardization, significant differences in the SUV_mean_ and SUV_max_ were found among the observers. Coregistering CT images with PET images improved the comparability to a limited extent. The introduction of a standardized protocol that details the VOI size and position for multiple organs reduced interobserver variability and enhanced comparability.

**Conclusions:**

The protocol offered clear guidelines and was particularly beneficial for beginners, resulting in improved comparability of SUV_mean_ and SUV_max_ values for various organs. The study suggested that incorporating an additional VOI template could further enhance the comparability of the findings in preclinical imaging analyses.

**Supplementary Information:**

The online version contains supplementary material available at 10.1007/s11307-024-01927-9.

## Introduction

Over the past few decades, preclinical molecular imaging, notably positron emission tomography (PET) combined with computed tomography (CT), has become indispensable in scientific medical research [1, 2]. This approach offers multimodal imaging in preclinical models that are highly translatable to clinical settings [3, 4]. PET enables quantification of biological processes in living subjects, achieved by defining regions or volumes of interest (ROIs or VOIs) on the images to extract activity concentrations (typically given in kBq/cc). Mathematical operations transform these activity concentrations into percent injected activity or dose per volume of tissue (%IA/cc or %ID/cc) by normalizing them to the administered activity or standardized uptake values (SUVs) by additionally normalizing to the body weight. The SUV is used as a semiquantitative measurement of glucose uptake in tissue from a 2-deoxy-2-[^18^F]fluoro-D-glucose ([^18^F]FDG) PET scan, especially in clinical practice [5]. The SUV_mean_, reflecting the mean voxel value within a VOI, is strongly influenced by the VOI definition method and is susceptible to partial volume effects, resulting in greater variability. Conversely, the SUV_max_, which represents the voxel with the highest radioactivity concentration, is less affected by observer variability but more affected by technical variations [6].

A major limitation in preclinical imaging is the lack of standardized or fully automated methods for defining VOIs. While some data-driven or semiautomatic segmentation methods exist, they still require observer input to define or choose the proposed cluster. Anatomy-based automatic segmentation methods rely heavily on annotated training images (magnetic resonance (MR) and/or CT), but their effectiveness hinges on the quality and quantity of the database. Currently, there is no widely accepted automated preclinical VOI delineation method. Consequently, most preclinical image analysis is manual, with observers selecting regions for analysis. Additionally, the availability of multiple software tools for preclinical PET/CT image analysis, each with different features and pipelines, further complicates the issue.

For clinical PET/CT imaging, several studies have assessed inter- and intraobserver variability and proposed methods to standardize image analysis [7–10]. Until now, there hasn't been any study conducted on preclinical PET/CT imaging that includes a standardized image analysis. Therefore, the present study assessed the variability in VOI size, SUV_mean,_ and SUV_max_ measurements of multiple organs and tumors between different observers (grouped into beginners and experts) when analyzing the same preclinical [^18^F]FDG-PET-only and [^18^F]FDG-PET/CT datasets with free or commercially available image analysis software. Furthermore, a standardized protocol was used, and all observers reanalyzed the PET/CT datasets following this protocol; potential improvements in interobserver variability were evaluated accordingly.

## Materials and Methods

### Imaging Data

Twelve observers analyzed dynamic [^18^F]FDG-PET-only (dynamic images 0–75 min, 25 frames; n = 6) and [^18^F]FDG-PET/CT (dynamic images 0–60 min, 19 frames; n = 7) scans of tumor-bearing mice. Two laboratories provided the datasets, which were acquired according to local regulations. The images were provided in Bq/cc together with the injected activities and weights of the mice in the scanner-specific and DICOM formats. Information regarding the animal experiments and imaging protocols can be found in the Electronic Supplementary Material (ESM). Co-registration of PET/CT data for part 2 and 3 was performed by one observer to eliminate potential co-registration-induced influences.

Of the twelve observers, eight were experts in the analysis of preclinical images (> 4 years of experience), whereas four were classified as beginners (< 1 year of experience). With the exception of the dataset providers, all observers analyzed the images independently and blinded to each other's assessments, utilizing their expertise and judgment.

## Part 1: [^18^F]FDG-PET-only Image Analysis and Reporting

The observers were asked to analyze the images according to their standard institutional procedures, including the choice of image analysis software, the procedures for preparing the images (e.g., adjustment of the animal's position), the radiation scale and time frames, and the method of delineating VOIs. The observers were requested to delineate the following VOIs: tumor, whole brain, muscle, heart (either whole heart or left ventricle), kidneys (left and right), liver, and urinary bladder (short name bladder). An additional region covering the whole FOV was delineated on the last time frame with a predefined size (128 × 128 × 95 voxels/51.2 × 51.2 × 75.62 mm^3^) to assess any software-related biases in image quantitation.

After analyzing the images, the observers completed a detailed report, including SUV_mean_ and SUV_max_ (normalized to the body weight of the animals, respectively), VOI delineation method (manual, thresholding, fixed objects, etc.), and volume (in mm3). They also specified how they displayed the images (radiation scale, minimum and maximum values, kBq/cc, %IA/cc, or SUV). As the datasets were dynamic, observers indicated the time frame (individual frame or summed image) for VOI delineation. Time-activity curves (TACs) for all animals and organs were plotted. Group differences (SUV_mean_ and SUV_max_) were determined across observers and animals based on the 10 min time frame from 55–65 min.

## Part 2: [^18^F]FDG-PET/CT Image Analysis and Reporting

The image analysis procedure for the PET/CT datasets was identical to that for the [^18^F]FDG-PET-only datasets. Only the whole FOV region was adjusted (256 × 256 × 159 voxels/99.377 × 99.377 × 126.564 mm^3^) as a different PET scanner was used for these experiments. In addition, the observers were asked to report on which dataset (PET or CT) each organ and the tumor were delineated. Group differences (SUV_mean_ and SUV_max_) were determined across observers and animals based on the 5 min time frame from 55–60 min.

## Part 3: Standardized [^18^F]FDG-PET/CT Image Analysis and Reporting

The authors established a standardized tumor and organ VOI definition method based on [^18^F]FDG-PET-only and [^18^F]FDG-PET/CT data analysis results. The protocol required to be universally applicable across image analysis software tools. Consequently, data-driven segmentation methods, such as multiclustering, were excluded from part 3, resulting in the exclusion of observer E8. Observer B3's analysis was also omitted due to inability to meet the standardized consensus specifications for VOI definition.

Observers unanimously opted to delineate organs and tumors using specific objects (ellipsoids and boxes), with predefined VOI drawing on either PET or CT images. PET-related VOIs adhered to a fixed radiation scale specified in SUV. VOIs for the brain, heart and tumor were delineated on the CT images (and verified on the respective PET images), as the CT image provided sufficient anatomical delineation to surrounding tissues. The VOIs for both muscle regions, kidneys, liver and both bladder regions were delineated on the PET images (and verified on the respective CT images) due to the fact that for most of these organs the [^18^F]FDG uptake is very distinct and the low soft-tissue contrast of the CT does not enable a clear delineation to surrounding tissues.

Table 1 summarizes the objects and predefined VOI sizes and ranges. To explore VOI position influence on quantitative analysis, two muscle regions (gluteus maximus and biceps/triceps) and two urinary bladder regions (bottom and maximum fill) were included.

**Table 1:** Details on the standardized VOI analysis. The PET-related VOIs were delineated at the last time frame using the specified SUV radiation scale

VOI	image used for VOI delineation	radiation scale (SUV)	shape	size	notes
tumor	CT	n.a	ellipsoid	entire tumor	
brain	CT	n.a	ellipsoid	7 × 5 × 10 mm^3^	inside skull, control on PET that olfactory bulb and harderian glands are excluded
heart	CT	n.a	ellipsoid	> 100 and < 200 mm^3^	
muscle	PET	0—2	box	2 × 2 × 3 mm^3^	gluteus maximus, avoid spill in from bladder, control on CT that no bone is included
muscle	PET	0—2	box	2 × 2 × 3 mm^3^	biceps/triceps, control on CT that no bone is included
kidney	PET	0—2	ellipsoid	~ 60 mm^3^	definition of right and left side
liver	PET	0—2	box	4 × 4 × 4 mm^3^	opposite to the stomach
bladder bottom	PET	0—10	box	2 × 2 × 2 mm^3^	bottom of bladder
bladder maximum fill	PET	0—10	ellipsoid	entire bladder	draw on time frame with largest bladder fill

### Statistical Analysis

The mean or maximum radioactivity concentrations given as SUV_mean_ or SUV_max_ per animal and organ over the 12 (part 1 and 2) and 10 (part 3) observers were used.

The coefficient of variation (CV, %) was calculated as the ratio of the standard deviation to the mean to assess the extent of variability. Moreover, to account for the variability between animals, the normalized difference was calculated for each animal and organ based on the 60 min values using the following equation:

$$normalized\;difference\;=\;\frac{individual\;value\;-\;mean\;value}{mean\;value}$$

The data are expressed as the mean ± standard deviation. Statistical analysis was performed with Prism 9.5.0 Software (GraphPad, La Jolla, CA, USA) and SPSS Statistics (version 29.0, IBM SPSS, IBM Corp., Armonk, NY, USA). Differences between the beginner and expert groups were assessed by applying two-way ANOVA followed by a Bonferroni multiple comparisons test, with an alpha level of 0.05 for each organ. Brown-Forsythe and Welch ANOVA tests were performed to assess interobserver variability, followed by Dunnett's multiple comparisons test, with individual variances computed for each comparison and organ. The threshold of statistical significance was set to an adjusted *p* value ≤ 0.05.

Intraclass correlation coefficients (ICCs; single-measure, two-way random, absolute agreement) were calculated based on the SUV_mean_ and SUV_max_ values to determine interobserver reliability for the beginners, the experts, and all observers [11, 12]. According to Koo et al. [12], ICCs less than 0.5 can be classified as poor reliability, ICCs in the range of 0.5 to 0.75 as moderate reliability, ICCs between 0.75 and 0.8 as good reliability, and ICCs greater than 0.9 as excellent reliability.

## Results

### Selection of Image Analysis Software Programs and VOI Definition Methods

Five different image analysis software programs were utilized in the present study. The selected software and the typically used output units, radiation scales, and time frames are summarized in the Suppl. Tab. s1 (see ESM). One observer employed a data-driven segmentation method (observer E8, BrainVISA/Anatomist) that used the local means analysis method based exclusively on the dynamics (i.e., time-activity and level of uptake) of each voxel in the PET images [13, 14]. The VOIs of six of the remaining eleven observers were defined in the last time frame. Some observers (3 out of 11) selected the time frame where the respective organ was clearly visible for analysis. Seven out of the eleven observers applied different radiation scales for specific organs (e.g., 0–2 SUV for muscle, 0–20 SUV for the heart), whereas the rest used a fixed radiation scale for all organs. The whole FOV region evaluated in parts 1 and 2 revealed no systematic software biases in image-based quantitation of the mean and maximum activity values (Suppl. Fig. s1**,** see ESM). These small differences were attributed to the VOI position in the whole FOV region.

## Parts 1 and 2: Individual [^18^F]FDG-PET-only and [^18^F]FDG-PET/CT Image Analysis

### VOI sizes

**Fig. 1:**
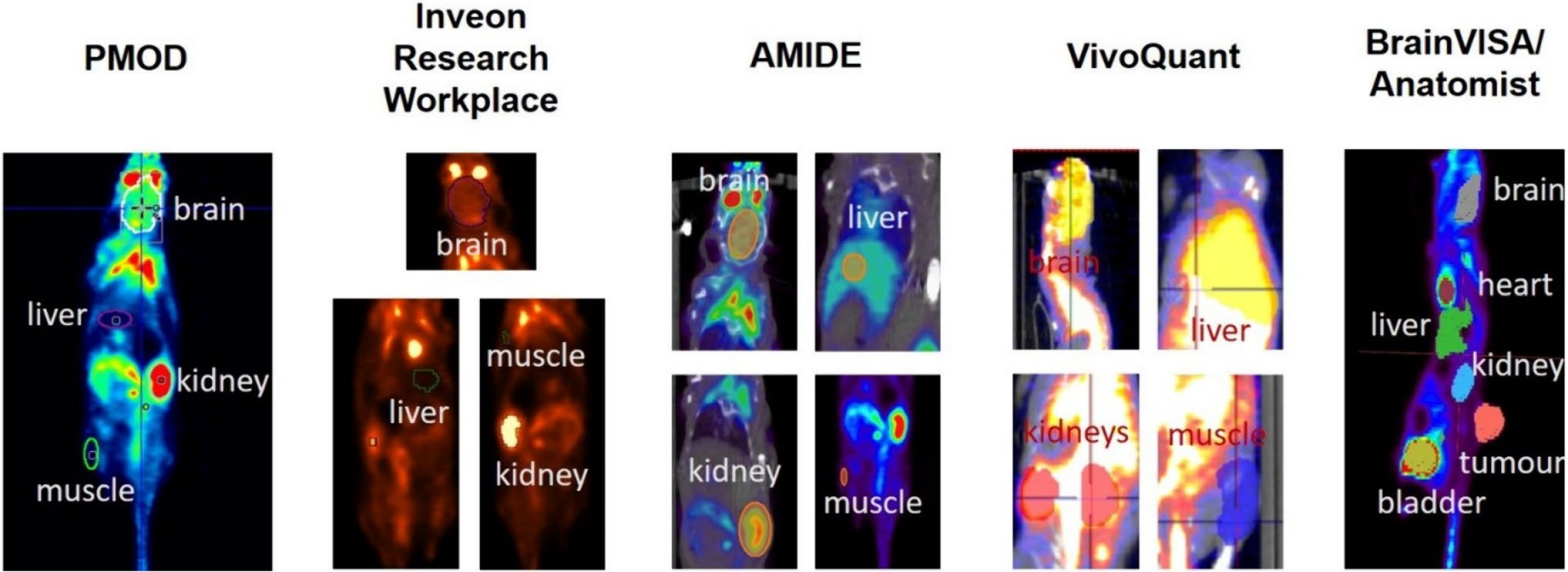
Representative images of multiple VOI positions for the individual software tools utilized for analysis. With the BrainVISA software, a 3D rendering of the VOIs is displayed.

The VOI delineation methods vary from fixed objects (e.g., spheres for the whole brain and heart) to manual drawings of VOIs on consecutive slices to those using thresholds (see Fig. 1 for examples of VOI positions and shape for each software tool). Some observers applied post-processing to re-orient the images according to the “standard” configuration in preclinical imaging (head first, prone), whereas others analyzed the images in the orientation provided by the scanner. The delineation methods used for each organ are summarized in the supplementary methods (Suppl. Fig. s2 and s3, see ESM) for the PET-only and PET/CT studies, respectively.

For the [^18^F]FDG-PET-only study, the tumor VOI was excluded from the analysis because delineation was rather challenging due to the low uptake and small size of the tumors (most of the observers could not identify the tumors).

**Fig. 2:**
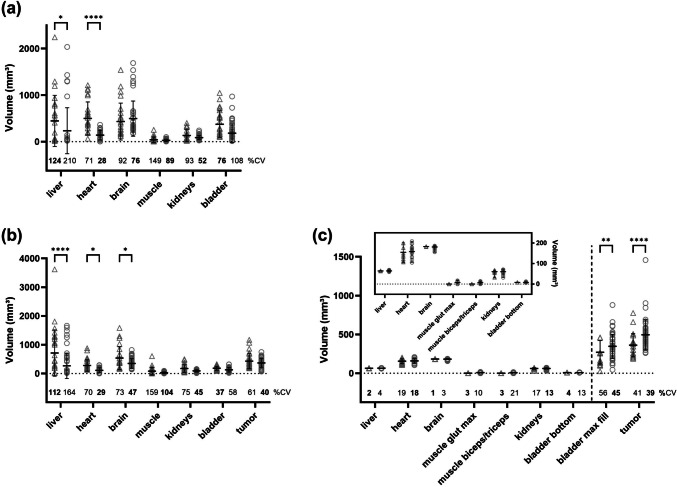
VOI sizes delineated by the beginner (n = 4, open triangle) or expert (n = 8, open circle) group on the **a** [^18^F]FDG-PET-only (n = 6) and **b** [^18^F]FDG-PET/CT (n = 7) images. In **c**, the VOI sizes after the standardization procedure are shown. The mean values ± standard deviations are displayed. (*p < 0.05; **p < 0.01; ***p < 0.001; ****p < 0.0001; two-way ANOVA followed by Bonferroni multiple comparisons test). The coefficient of variation (%CV) values for each organ are provided separately for beginners and experts. The bold text marks lower %CV values for beginners or experts. (Abbreviations used: bladder – urinary bladder, muscle glut max – muscle gluteus maximus, bladder bottom – bottom of the urinary bladder, bladder max fill – urinary bladder at maximum fill).

The different delineation methods resulted in considerable variability in the VOI sizes, as illustrated in Fig. 2(a) [^18^F]FDG-PET-only; (b) [^18^F]FDG-PET/CT). The beginners delineated significantly larger liver and heart VOIs than did the experts on the PET images (part 1). The smallest variability in the VOI sizes in the beginner group was obtained for the heart (71% CV), whereas in the expert group, the smallest variability was obtained for the kidneys (52% CV). In contrast, the greatest variability was found in the muscle VOI (149% CV) for the beginner group and in the liver VOI (210% CV) for the expert group.

On the [^18^F]FDG-PET-CT images (part 2), the beginners delineated significantly larger VOIs than did the experts in the liver, heart, and brain. The smallest variability in VOI sizes was obtained in the bladder for the beginners (37% CV) and in the tumor VOIs for the experts (40% CV). The highest variability in VOI sizes was found in the muscle for the beginners (159% CV) and in the liver for the experts (164% CV). In particular, the VOI drawn for the liver ranged from 16 to 3619 mm3, which spans two orders of magnitude. Furthermore, the VOI position for the muscle differed among the observers (e.g., for part 2, the lower left limb was delineated by seven observers, the upper left limb was delineated by four observers, and the upper right limb was delineated by one observer).

### Organ-time activity curves

The organ TACs for part 1 [^18^F]FDG-PET-only images for a representative animal, subdivided into beginner and expert groups, are shown in Suppl. Fig. s4 (SUV_mean_) and Fig. s5 (SUV_max_) in the ESM. The heart and kidney SUV_mean_ TACs exhibited greater interobserver variation in the beginner group than in the expert group. The remaining organs revealed a similar pattern between beginners and experts.

For the SUV_max_ of the TACs, the beginner group revealed greater interobserver variation for the brain and muscle; interestingly, the experts showed greater variability than the beginners for the liver and heart.

The inclusion of CT data (part 2) reduced the variability in the liver, brain, and muscle SUV_mean_ TACs, as depicted in Suppl. Fig. s6 and Fig. s7 (see ESM). For the SUV_max_ of the TACs (beginners: Suppl. Fig. s8; experts: Suppl. Fig. s9), reduced variability was detected mainly for the muscle. The two groups of observers determined identical SUV_max_ TACs for the tumor, kidney, and bladder.

### Last time frame analysis

**Fig. 3:**
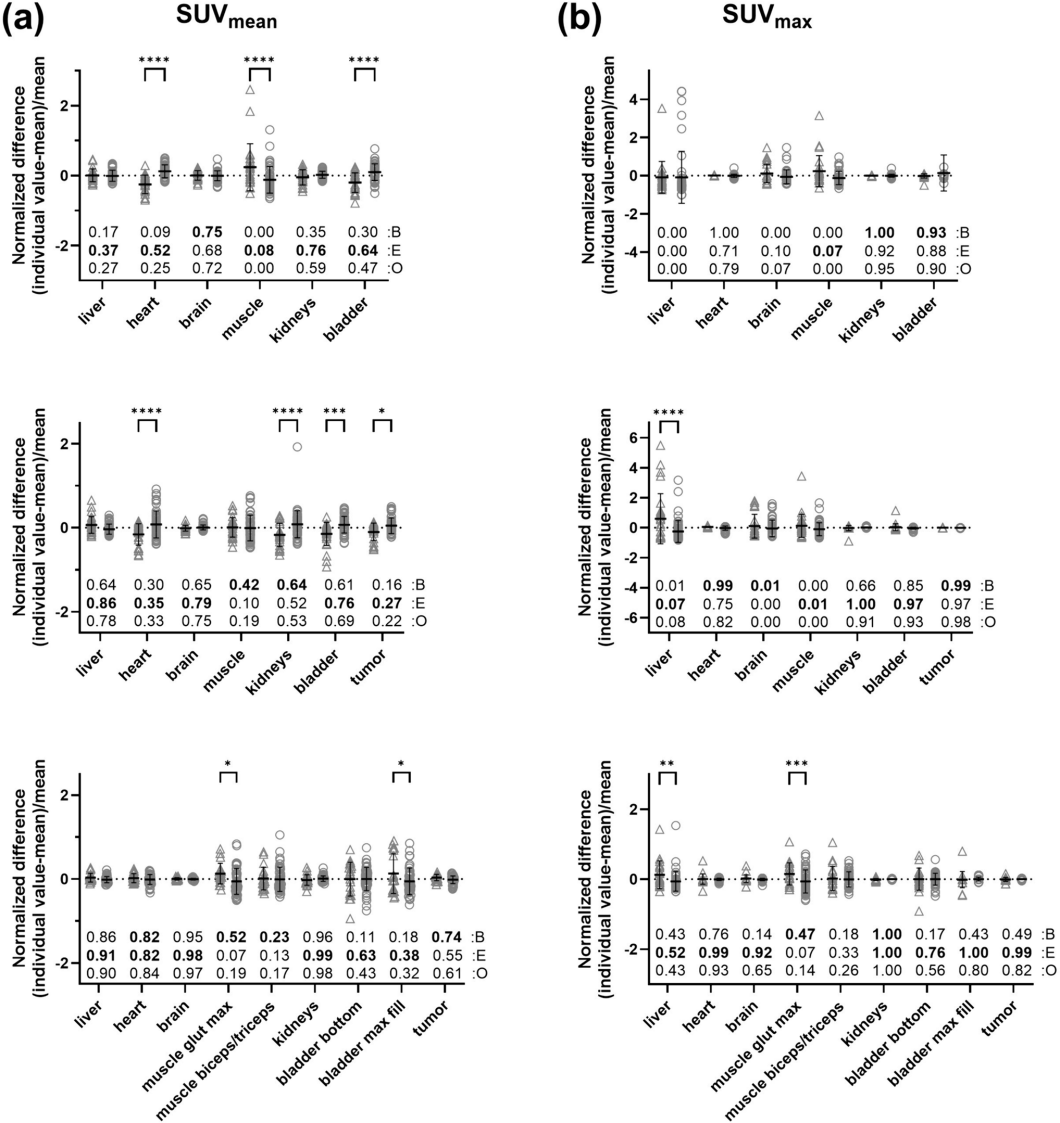
**a** SUV_mean_ and **b** SUV_max_ analysis for the different organs for [^18^F]FDG-PET-only (upper row), [^18^F]FDG-PET/CT (middle row) and standardized [^18^F]FDG-PET/CT (lower row) analysis by beginners (n = 4/3, open triangle) and experts (n = 8/7, open circle). The normalized difference for each animal is plotted. The mean values ± standard deviations are displayed. (*p < 0.05; **p < 0.01; ***p < 0.001; ****p < 0.0001; two-way ANOVA followed by Bonferroni multiple comparisons test). The ICCs for each organ are provided separately for beginners (B), experts (E), and all observers (O). A bold text indicates greater reliability for beginners or experts. (Abbreviations used: bladder – urinary bladder, muscle glut max – muscle gluteus maximus, bladder bottom – bottom of the urinary bladder, bladder max fill – urinary bladder at maximum fill).

The SUV_mean_ and SUV_max_ values from the time frame covering 60 min were used to compare the variability between groups (beginners and experts) and individual observers. For the PET-only study, the calculated normalized difference based on the SUV_mean_ showed the greatest deviation from 0 for the heart region (-0.25 ± 0.27 for beginners and 0.13 ± 0.18 for experts) and the smallest deviation for the brain (0.01 ± 0.14 for beginners and -0.01 ± 0.14 for experts), as displayed in the upper row of Fig. 3(a). In addition, statistically significant differences were observed between the beginner and expert groups for the heart, muscle and bladder. The ICCs revealed greater reliability within the expert groups for all organs except the brain, although poor reliability was observed for the muscle and liver (ICCs < 0.5).

The calculated normalized difference based on the SUV_max_ (Fig. 3(b)) yielded the greatest deviation from 0 for the muscle region among the beginners (0.24 ± 0.81) and for the bladder among the experts (0.14 ± 0.95). The smallest deviation was found for the kidney region (beginners: 0.01 ± 0.02; experts: -0.01 ± 0.07). Overall, no statistically significant differences between the observer groups were observed. An overview of all the ICCs, including confidence intervals (CIs), for each organ can be found in the supplementary materials (Suppl. Tab. s2, see ESM).

**Fig. 4:**
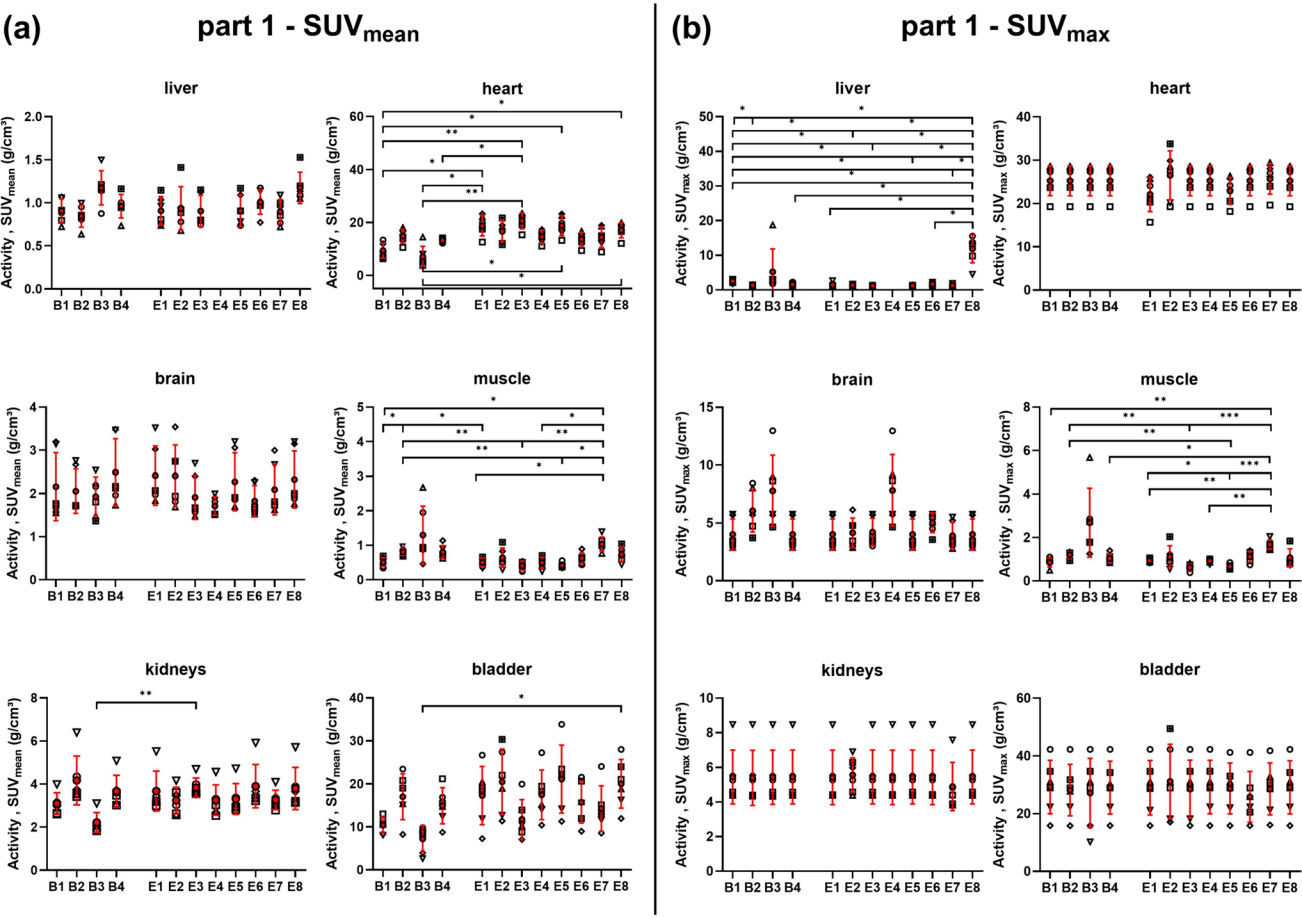
**a** SUV_mean_ and **b** SUV_max_ analysis as a function of beginner or expert observers for [^18^F]FDG-PET-only data from the liver, heart, brain, muscle, mean kidney, and urinary bladder. Individual values, as well as the mean ± standard deviation, are displayed. B1-4: beginners 1 to 4; E1-8: experts 1 to 8. Differences between individual observers were assessed by Brown-Forsythe and Welch ANOVA followed by Dunnett’s T3 multiple comparisons test (*p < 0.05; **p0 < 0.01; ***p < 0.001; ****p < 0.0001). Expert 4 did not analyze the liver. (Abbreviations used: bladder – urinary bladder).

Multiple statistically significant differences in the SUV_mean_ were detected between the individual observers, especially for the heart and muscle VOIs_,_ as shown in Fig. 4(a). For the SUV_max_, the liver and muscle indices revealed multiple significant differences among the 12 observers (Fig. 4(b)). The individual p values are given in Suppl. Fig. s10 (see ESM).

For the PET/CT study, the normalized difference of the muscle for beginners and experts was reduced (compare the middle row of Fig. 3(a)). However, statistically significant differences between the observer groups were obtained for the heart, kidneys, bladder, and tumor. The ICCs for the liver, muscle, and bladder showed improved reliability compared to those of part 1. Analyzing the normalized difference based on the SUV_max_ (Fig. 3(b)) yielded the largest overall spread in the liver region (0.60 ± 1.67 for the beginners and -0.25 ± 0.73 for the experts, p < 0.0001). No improvement in reliability was detected for the ICCs based on the SUV_max_ for part 2 compared to part 1.

**Fig. 5:**
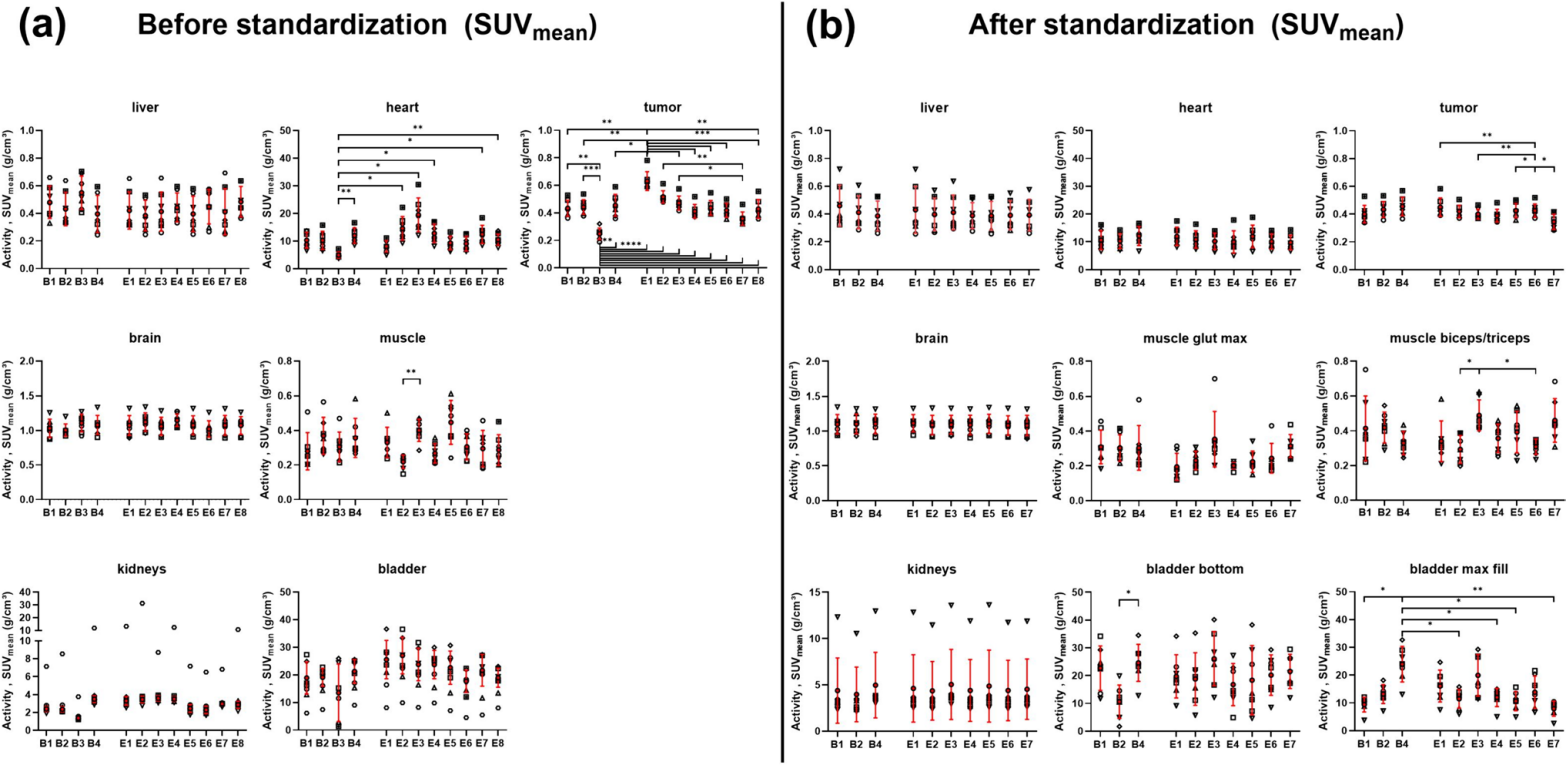
SUV_mean_ analysis as a function of beginner or expert observers from [^18^F]FDG-PET/CT data for the selected regions **a** before and **b** after standardization. Individual values, as well as the mean ± standard deviation, are displayed. B1-4: beginners 1 to 4; E1-8: experts 1 to 8. Differences between individual observers were assessed by Brown-Forsythe and Welch ANOVA followed by Dunnett’s T3 multiple comparisons test (*p < 0.05; **p < 0.01; ***p < 0.001; ****p < 0.0001). The analyses of observers B3 and E8 were not included in the standardized [^18^F]FDG-PET/CT analysis because they were not applicable for the standardized protocol. (Abbreviations used: bladder – urinary bladder, muscle glut max – muscle gluteus maximus, bladder bottom – bottom of the urinary bladder, bladder max fill – urinary bladder at maximum fill).

**Fig. 6:**
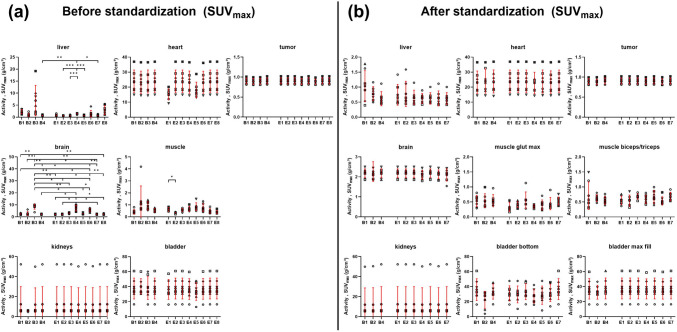
SUV_max_ analysis as a function of beginner or expert observers from [^18^F]FDG-PET/CT data for the selected regions **a** before and **b **after standardization. Individual values, as well as the mean ± standard deviation, are displayed. B1-4: beginners 1 to 4; E1-8: experts 1 to 8. Differences between individual observers were assessed by Brown-Forsythe and Welch ANOVA followed by Dunnett’s T3 multiple comparisons test (*p < 0.05; **p < 0.01; ***p < 0.001; ****p < 0.0001). The analyses of observers B3 and E8 were not included in the standardized [^18^F]FDG-PET/CT analysis because they were not applicable for the standardized protocol. (Abbreviations used: bladder – urinary bladder, muscle glut max – muscle gluteus maximus, bladder bottom – bottom of the urinary bladder, bladder max fill – urinary bladder at maximum fill).

The interobserver SUV_mean_ and SUV_max_ variability are displayed in Fig. 5(a) and 6(a), revealing multiple statistically significant differences in the heart and tumor regions (both SUV_mean_) as well as the liver and brain regions (both SUV_max_). The individual p values between the observers are given in Suppl. Fig. s11 and Fig. s12 (see ESM).

## Part 3: standardized [^18^F]FDG-PET/CT image analysis

The predefined VOI sizes reduced the variations, as shown in Fig. 2(c). However, for the two regions for which the entire structure was to be delineated, namely, the tumor and the bladder at the maximum-fill level, significantly larger VOIs were determined by experts with great variability (tumor: beginners: 41% CV; experts: 38% CV; bladder: beginners: 56% CV; experts: 45% CV).

### Organ-time activity curves after standardization

The standardized image analysis method reduced the variation in the SUV_mean_ TACs of the tumor, brain, liver, and kidney, as shown in panel B in the Suppl. Fig. s6 and s7 (see ESM). The muscle and bladderTACs exhibited different patterns depending on the VOI position. The expert group obtained mostly congruent SUV_max_ TACs for the liver, heart, tumor, brain, kidneys, and bladder maximum-fill VOIs (Suppl. Fig. s9), whereas the beginner group obtained slightly greater variations (Suppl. Fig. s8, see ESM).

### Last time frame analysis after standardization

The standardized analysis approach notably enhanced the normalized difference based on SUV_mean_ for most organs, depicted in the lower row of Fig. 3(a), correlating with higher ICCs across most organs. Liver and brain index reliability significantly improved, achieving excellent levels post-standardization. Initially poor heart and tumor reliability transformed into good and moderate levels, respectively. Standardization notably elevated kidney index reliability from moderate to excellent levels. However, statistically significant differences persisted between observer groups for muscle gluteus maximus and urinary bladder maximum-fill regions. Improvement in normalized difference based on SUV_max_ was inconsistent post-standardization, with no improvement observed for tumor or urinary bladder (Fig. 3(b)). Significant differences between observer groups were found for liver and gluteus maximus region (SUV_max_). Notably, liver and brain ICCs substantially improved in standardized analysis (liver: part 2 = 0.08, part 3 = 0.43; brain: part 2 = 0.00, part 3 = 0.65).

The interobserver variability based on the SUV_mean_ values was markedly reduced using the standardized image analysis approach. However, some statistically significant differences between observers persisted in the tumor, biceps/triceps muscle, or maximum-fill urinary bladder region (Fig. 5(b)).). The individual p values between the observers are given in Suppl. Fig. s13 (see ESM). For the SUV_max_, no significant differences were found between the observers for any of the organs (Fig. 6(b)).

## Discussion

Quantifying radioactivity concentrations in small animal organs or tumors is standard in preclinical imaging and relies on parameters such as the SUV_mean_ or SUV_max_. However, the variability and reproducibility of these parameters among different observers within a single institution or across multiple centers remain poorly understood. Currently, each imaging lab and often each observer within the same institution applies different workflows, experiences, and judgments to analyze and segment PET images. These variations encompass factors such as the position, size, and shape of VOIs; PET image display settings; and postprocessing methods, potentially compromising comparability across observers and centers. Despite the prevalence of preclinical [^18^F]FDG-PET/CT studies, no multicenter consensus exists on a reproducible image analysis method. This study represents the first comprehensive multicenter [^18^F]FDG-PET/(CT) investigation into the impact of image analysis methods on results and the comparability of a standardized analysis approach. Our findings underscore the significant influence of image analysis methods on [^18^F]FDG-PET/(CT) study outcomes, particularly regarding SUV_mean_ discrepancies attributed to regional position and size, corroborating similar observations from prior studies [15].

Our first observation was that not all observers performed post-processing to re-orient the images according to the “standard” configuration in preclinical imaging (head first, prone). Some analyzed the images in the orientation provided by the scanner, which was for the PET/CT study in feet first, prone. Thus, an agreement on the orientation of images to be used (also with regard to future automatic segmentation applications) is therefore the first step towards standardized image analysis. Without standardization, variations in VOI sizes were observed between beginners and experts for multiple organs. These differences influenced SUV_mean_ (e.g., heart) and SUV_max_ (e.g., liver in PET/CT) analyses, suggesting that VOI size impacts uptake. However, for certain organs (e.g., the liver in PET-only and the brain in PET/CT), despite significant differences in VOI size, SUV analysis was unaffected by homogeneous [^18^F]FDG uptake.

Introducing anatomical references in part 2 reduced variability in heart and muscle regions but had no effect on liver or brain regions. However, overall reliability and comparability did not improve universally. Comparing parts 1 and 2 is challenging due to the different image sets analyzed. However, this design showcases variability between studies (e.g., small vs. large tumors with necrotic areas), mitigating potential biases from part 1 to part 2.

Based on the results from these two studies, the participants in this study reached a consensus on the standardized VOI delineation method utilized in part 3.

Standardization improved the consistency and shape of SUV_mean_ TACs in the liver, brain, and kidney, while nearly identical SUV_max_ TACs were obtained in the liver, heart, tumor, brain, kidneys, and urinary bladder. Reduced interobserver variability poststandardization was evidenced by reduced deviation and improved ICCs across organs, except for muscle and urinary bladder regions. Muscle VOIs are small and prone to spill over from adjacent bone regions, making muscle-fat differentiation challenging despite the use of anatomical information from CT scans. Intensive training and visual aids are recommended for comparability improvement. For maximum-fill bladder VOIs, inconsistent time frame choices hindered comparisons between parts 2 and 3. Nevertheless, considering its importance in dosimetric studies, assessing bladder necessity and employing frame-by-frame analysis for volumetric changes are advised.

Furthermore, the significant differences between beginners and experts found by the normalized difference analysis in the heart, kidneys, and tumor diminished after standardization (Fig. 3(b) and 3(c)). We concluded that the use of a standardized approach reduced the interobserver variability in the SUV analysis. In addition, we propose to create a VOI template for each preclinical PET/CT and PET/MR study that includes a standardized VOI positioning and size as well as detailed information on the segmentation method. For multicenter studies, we recommend reaching a consensus on the use of single analysis software for evaluating and providing VOI template files. For single-center studies, a VOI template from the first animal analyzed will ensure reproducibility for the remaining animals and help train new personnel.

In general, the SUV_max_ revealed a lower interobserver variability than the SUV_mean_ in our study. However, as the SUV_max_ represents only a single voxel within a region, the SUV_mean_ might be a more stable marker for underlying tissue uptake. Therefore, both measures can be valuable in multicenter studies.

Despite its strengths, our study has several limitations. First, mid-level observers were not included, potentially biasing the results, as experience levels were subjectively categorized as beginners or experts. Additionally, the varied backgrounds of the participating observers (e.g., physics, chemistry, biology, etc.) may have influenced interpretation. Secondly, validation using gamma-counter data was not available. Third, the use of different image analysis software led to the use of various segmentation tools, hindering detailed discrepancy identification within segmented VOIs. Finally, the standardized protocol lacked optimization, notably omitting a VOI template for precise location visualization. Addressing these limitations in future studies could enhance the accuracy and reproducibility of the findings.

It has to be noted that depending on the specific tracer used, standardized image analysis protocols need to be re-defined to address tracer-specific factors that might impact the reproducibility of image analysis. This also applies for the acquisition of the imaging data, for which standardized protocols – depending on the used tracer – can also significantly enhance reproducibility [16].

The 12 observers in this study represent 8 different preclinical imaging facilities in Europe and all observers were asked to use their default image analysis method and software tool to analyze the provided PET(/CT) data. Only 1 observer analyzed the data using an automated segmentation tool. Automatic organ segmentation has been an active field of research for decades [17–22], and current research in this field includes the development of artificial intelligence (AI)-assisted solutions [23]. Nevertheless, manual delineation will still be the standard method for image analysis until these tools are applicable to a broader community with sufficient training databases and a variety of VOI templates. The variety of chosen software tools and methods utilized in this study encompasses, in our opinion, the most used methods in image analysis in preclinical PET imaging. However, the transition to AI-guided automatic segmentation will certainly be a strong focus within the next decade and thus will potentially improve the comparability and reliability of preclinical multicenter image analysis.

## Conclusion

For the first time, the present study demonstrated the significant influence of image analysis on the obtained quantitative data; this work is intended as the basis for a discussion of further standardization approaches in preclinical imaging. Moreover, the authors aim to raise awareness of potential pitfalls when preclinical data are analyzed by multiple observers with different levels of experience. Our study verified that the comparability of image analysis significantly improves when detailed standardized image analysis protocols are used. This approach will be of particular interest not only for preclinical multicenter studies but also for studies performed over a long period within the same institution, where the observers might vary.

### Supplementary Information

Below is the link to the electronic supplementary material.

Supplementary file1 (DOCX 6461 KB)

## Data Availability

The authors declare that the data supporting the findings of this study are available within the paper and its Supplementary Information file. Should any raw data files be needed in another format they are available from the corresponding author upon reasonable request.
