# Supplementary material for: Optimizing SUV Analysis: A Multicenter Study on Preclinical FDG-PET/CT Highlights the Impact of Standardization
Source: Mol Imaging Biol. 2024 Jun 21;26(4):668–79. doi: 10.1007/s11307-024-01927-9 (PMC11281957; doi:10.1007/s11307-024-01927-9)
Supplement: Supplementary file 1 — Supplementary file1 (DOCX 6461 KB) [file 11307_2024_1927_MOESM1_ESM.docx]

**Electronic Supplementary Material**

***Optimizing SUV Analysis: Multicenter Study on Preclinical FDG-PET/CT Highlights the Impact of Standardization***

**Journal: Molecular Imaging and Biology**

Claudia Kuntner^1, 2^, Carlos Alcaide^3^, Dimitris Anestis^4^, Jens Bankstahl^5^, Herve Boutin^6^, David Brasse^7^, Filipe Elvas^8^, Duncan Forster^6^, Maritina G. Rouchota^4^, Adriana Tavares^3^, Mari Teuter^5^, Thomas Wanek^1^, Lena Zachhuber^1^, Julia G. Mannheim^9, 10^

^1^ Medical University of Vienna, Vienna, Austria

^2^ Medical Imaging Cluster (MIC), Vienna, Austria

^3^ University of Edinburgh, Edinburgh, United Kingdom

^4^ BIOEMTECH, Athens, Greece

^5^ Hannover Medical School, Hannover, Germany

^6^ University of Manchester, Manchester, United Kingdom

^7^ Université de Strasbourg, Strasbourg, France

^8^ University of Antwerpen, Antwerpen, Belgium

^9^ Werner Siemens Imaging Center, Department of Preclinical Imaging and Radiopharmacy, Eberhard-Karls University Tuebingen, Tuebingen, Germany

^10^ Cluster of Excellence iFIT (EXC 2180) “Image Guided and Functionally Instructed Tumor Therapies”, Tuebingen, Germany

Corresponding & first author: Claudia Kuntner

Address: Waehringer Guertel 18-20, 1090 Vienna, Austria

E-mail address: claudia.kuntner@meduniwien.ac.at

Telephone number: +43 (0)1 40160-64022

Material and Methods

[^18^F]FD PET-only (part 1)

Tumor model generation

Female 8-week-old athymic Crl:NU(NCr)-Foxn1nu mice were obtained from Charles River (Sulzfeld, Germany) to generate the breast tumor model. At the time of the experiment, the animals weighed 25.6 ± 2.8 g. One week before tumor cell inoculation, an estrogen pellet (17β-ESTRADIOL, 1.7 mg/pellet, 60 days release, pellet size: 3.0 mm, Innovative Research of America, Sarasota, FL, USA) was implanted into the left shoulder region under isoflurane anesthesia and aseptic conditions. The human breast cancer cell line MCF7 (ATCC® HTB-22™) was obtained from the American Type Culture Collection (ATCC, Manassas, VA, USA) and grown as an adherent culture in a base medium containing 0.01 mg/mL human recombinant insulin, 10% FBS, and 1% Pen-Strep. MCF7 cells were harvested, suspended in serum-free RPMI-1640 medium and L-glutamine, and 5-8 × 10^6^ cells in a volume of approximately 100 μL were subcutaneously injected into the right upper shoulder of the animals. The national authorities approved all animal experiments (Amt der Niederösterreichischen Landesregierung, GZ: LF1-TVG-48/022-2016), and all study procedures were performed in accordance with the European Communities Council Directive of September 22, 2010 (2010/63/EU). All efforts were made to comply with the 3Rs principle in this study.

Imaging procedure

For the PET scans, the animals were first anesthetized in an induction chamber using isoflurane (4-5% in air) and then transferred to a double imaging chamber. A microPET Focus 220 scanner (Siemens Medical Solutions, Knoxville, TN) was used for PET imaging. Before radiotracer injection, a 10-min transmission scan using a rotating 57Co source was recorded. For the acquisition of the dynamic PET scans, an energy window of 250-750 keV and a timing window of 6 ns were used. During the scans, the respiratory rate and body temperature of the animals were constantly monitored (SA Instruments Inc., Stony Brook, NY, USA), and the isoflurane level was adjusted (1.5-2.5% in air) to achieve a constant depth of anesthesia. Approximately 20 ± 8 days after inoculation, the tumors had reached a size of 100-200 mm^3^, and PET imaging was performed. Mice were fasted for 6 h prior to [^18^F]FDG injection. Thereafter, a catheter was positioned in the tail vein, and a 75-min dynamic PET scan was started simultaneously with [^18^F]FDG injection (6.6 ± 1.8 MBq).

Dynamic [^18^F]FDG-PET emission data were sorted into 25 time frames, which incrementally increased in time length from 5 s to 10 min. All PET images were reconstructed using Fourier rebinning of the 3D sinograms followed by two-dimensional filtered back projection with a ramp filter, resulting in a voxel size of 0.4 x 0.4 x 0.798 mm^3^. The standard data correction protocol (normalization, attenuation, and decay correction) was applied to the PET data.

[^18^F]FDG-PET/CT (part 2 and 3)

Tumor model generation

Seven 12 week-old CBA nude mice were bred in house by a biological service facility in Manchester and implanted intradermally with 0.1 mL of a 5 x 10^7^/ml suspension of colorectal cancer HCT116 cells on the back of the animals. When tumor sizes reached ~200 mm^3^ at approximately day 18 after implantation, animals were transferred to the imaging facility. The local authorities approved all animal experiments.

Imaging procedure

When tumor sizes reached ~200 mm^3^ at approximately day 18 after implantation, animals were transferred to the imaging facility. Mice underwent 60-min dynamic [^18^F]FDG-PET/CT scans using an Inveon MultiModality PET/CT scanner (Siemens Medical Solutions, Knoxville, TN) on day 17 and day 20 after baseline scans and radiotherapy doses of 2 Gy on day 1 to 5 and day 8 to 12. For the PET/CT scans, mice were anesthetized in an induction chamber (isoflurane with oxygen at 2L/min) and subsequently transferred to an anesthetic facemask on a heated bed and settled for 5 minutes, while the tail vein was dilated and catheterized for *i.v.* injection. Mice were then transferred to the temperature-controlled imaging bed and attached to an anesthetic facemask. CT scans (voltage 80 kVp, current 500 µA, exposure time 230 ms, 121 projections, binning 4, matrix size 480 x 480 x 639, pixel size 0.204941 mm^3^) were performed pre-injection of the tracer. Afterward, 10-15 MBq of [^18^F]FDG (<200 µL) was injected and PET scans commenced immediately after tracer injection. Dynamic PET data was histogrammed in 19 frames (5 x 60 s, 5 x 120 s, 9 x 300 s) and reconstructed using an ordered subset expectation maximization (OSEM3D) algorithm with 4 iterations and 16 subsets and a matrix size of 256 resulting in a reconstructed voxel size of 0.0388192 x 0.038819 x 0.0796 mm^3^. The in-house default correction methods (normalization, attenuation, scatter, dead-time, decay correction) were applied to the PET data.

**Tab s1** Summary of the observers, output units, used radiation scales, time frames for VOI definition, choice of software, and experience level. The radiation scale and selected time frames are based on parts 1 and 2.

| **observers** (E: expert; B: beginner) | **output units** | **set radiation scale** | **PET**  **time frame (min), part 1** | **PET/CT**  **time frame (min), part 2** | **software** |
| --- | --- | --- | --- | --- | --- |
| B1 | kBq/ml | 0-2000 kBq/ml | 55-65 | 55-60 | PMOD (version 4.0, PMOD Technologies LLC Zurich, Switzerland) |
| B2 | %IA/cc | *0-10 %IA/cc** | 65-75 | 30-60 | PMOD (version 4.3) |
| B3^+^ | Bq/cc | 0-70% of maximum intensity* | 1-5^#^ | 35-60^#^ | VivoQuant (version 4.0, Invicro, Needham, MA, USA)^+^ |
| B4 | SUV | 0-3 SUV* | 7.5-65^#^ | 7.5-55^#^ | AMIDE (version 1.0.6, <https://amide.sourceforge.net/>)(*11*) |
| E1 | %IA/cc | *0-10 %IA/cc** | 45-75^#^ | 0-60^#^ | AMIDE (version 1.0.4) |
| E2 | SUV | >40% max | 65-75 | 55-60 | AMIDE (version 1.0.5) |
| E3 | %IA/cc | 0-1000 kBq/ml | 65-75 | 55-60 | Inveon Research Workplace (version 4.2, Siemens Medical Solutions, Knoxville, TN) |
| E4 | %IA/cc | 0-500 kBq/ml | 65-75 | 55-60 | PMOD (version 3.611) |
| E5 | SUV | *0-5 SUV** | 0-75 | 0-60 | AMIDE (part 1 & 2: version 1.0.4, part 3: version 1.0.6) |
| E6 | %IA/cc | *0-10 %IA/cc** | 65-75 | 55-60 | PMOD (version 4.3) |
| E7 | SUV | *0-5 SUV** | 65-75 | 55-60 | Inveon Research Workplace (version 4.2) |
| E8^§^ | Bq/cc | N/A | 0-75 | 0-60 | BrainVISA/Anatomist (version 4.1.1, <https://brainvisa.info/web/>)^§^ |

**the observers adapted the radiation scale based on the radioactivity concentration in the respective organ*

*^#^the observers selected the time frame in which the respective organ was clearly visible (e.g., for the liver, an early time frame was used, whereas, for the urinary bladder, a later time frame was used)*

^§^*data-driven segmentation analysis; not included in the standardized* *[^18^F]FDG-PET/CT analysis (part 3); N/A=not applicable*

^+^*not included in the standardized [^18^F]FDG-PET/CT analysis (part 3) as the utilized software tool did not enable the definition of VOIs as required by the standardized manual*

**Tab s2** ICCs and corresponding confidence intervals for each organ separately listed for experts, beginners and all observers for part 1, 2 and 3 and SUV_mean_ and SUV_max_. Negative ICC values were set to 0.

| **part 1** | SUV_mean_ | | | SUV_max_ | | |
| --- | --- | --- | --- | --- | --- | --- |
|  | ICC (CI lower bound/upper bound) | | | ICC (CI lower bound/upper bound) | | |
|  | beginners | experts | all observers | beginners | experts | all observers |
| liver | 0.17 (-0.04/0.66) | 0.37 (0.11/0.81) | 0.27 (0.08/0.73) | 0.00 (-0.21/0.51) | 0.00 (-0.02/0.06) | 0.00 (-0.02/0.14) |
| heart | 0.09 (-0.04/0.52) | 0.52 (0.21/0.88) | 0.25 (0.08/0.69) | 1.00 (1.00/1.00) | 0.71 (0.42/0.94) | 0.79 (0.56/0.96) |
| brain | 0.75 (0.38/0.95) | 0.68 (0.37/0.93) | 0.72 (0.45/0.94) | 0.00 (-0.12/0.45) | 0.10 (-0.01/0.52) | 0.07 (-0.01/0.42) |
| muscle | 0.00 (-0.22/0.30) | 0.08 (-0.01/0.44) | 0.00 (-0.04/0.15) | 0.00 (-0.14/0.33) | 0.07 (-0.01/0.41) | 0.00 (-0.04/0.12) |
| kidneys | 0.35 (0.03/0.81) | 0.76 (0.48/0.95) | 0.59 (0.29/0.90) | 1.00 (1.00/1.00) | 0.92 (0.80/0.99) | 0.95 (0.87/0.99) |
| urinary bladder | 0.30 (0.02/0.77) | 0.64 (0.33/0.92) | 0.47 (0.20/0.85) | 0.93 (0.79/0.99) | 0.88 (0.71/0.98) | 0.90 (0.77/0.98) |

| **part 2** | SUV_mean_ | | | SUV_max_ | | |
| --- | --- | --- | --- | --- | --- | --- |
|  | ICC (CI lower bound/upper bound) | | | ICC (CI lower bound/upper bound) | | |
|  | beginners | experts | all observers | beginners | experts | all observers |
| liver | 0.64 (0.22/0.91) | 0.86 (0.67/0.97) | 0.78 (0.55/0.95) | 0.01 (-0.11/0.40) | 0.07 (-0.02/0.38) | 0.02 (-0.02/0.24) |
| heart | 0.30 (0.02/0,74) | 0.35 (0.11/0.75) | 0.33 (0.12/0.72) | 0.99 (0.98/1.00) | 0.75 (0.44/0.94) | 0.82 (0.59/0.96) |
| brain | 0.65 (0.30/0.91) | 0.79 (0.56/0.95) | 0.75 (0.52/0.94) | 0.01 (-0.02/0.19) | 0.00 (-0.02/0.09) | 0.00 (-0.01/0.06) |
| muscle | 0.42 (0.08/0.82) | 0.10 (0.00/0.46) | 0.19 (0.05/0.58) | 0.00 (-0.19/0.43) | 0.01 (-0.05/0.25) | 0.00 (-0.05/0.15) |
| kidneys | 0.64 (0.22/0.91) | 0.52 (0.25/0.86) | 0.53 (0.28/0.85) | 0.66 (0.32/0.92) | 1.00 (1.00/1.00) | 0.91 (0.79/0.98) |
| urinary bladder | 0.61 (0.25/0.90) | 0.76 (0.50/0.94) | 0.69 (0.43/0.92) | 0.85 (0.62/0.97) | 0.97 (0.93/0.99) | 0.93 (0.85/0.99) |
| tumor | 0.16 (0.00/0.56) | 0.27 (0.07/0.68) | 0.22 (0.07/0.60) | 0.99 (0.93/1.00) | 0.97 (0.89/0.99) | 0.98 (0.93/1.00) |

| **part 3** | SUV_mean_ | | | SUV_max_ | | |
| --- | --- | --- | --- | --- | --- | --- |
|  | ICC (CI lower bound/upper bound) | | | ICC (CI lower bound/upper bound) | | |
|  | beginners | experts | all observers | beginners | experts | all observers |
| liver | 0.86 (0.46/0.97) | 0.91 (0.79/0.98) | 0.90 (0.76/0.98) | 0.43 (0.03/0.84) | 0.52 (0.23/0.86) | 0.43 (0.19/0.80) |
| heart | 0.82 (0.45/0.96) | 0.82 (0.60/0.96) | 0.84 (0.64/0.96) | 0.76 (0.37/0.95) | 0.99 (0.97/1.00) | 0.93 (0.83/0.99) |
| brain | 0.95 (0.83/0.99) | 0.98 (0.94/1.00) | 0.97 (0.92/0.99) | 0.14 (-0.30/0.72) | 0.92 (0.80/0.98) | 0.65 (0.40/0.91) |
| muscle glut max | 0.52 (0.01/0.88) | 0.07 (-0.04/0.44) | 0.19 (0.04/0.59) | 0.47 (-0.05/0.87) | 0.07 (-0.06/0.47) | 0.14 (0.01/0.54) |
| muscle biceps/triceps | 0.23 (-0.16/0.75) | 0.13 (-0.01/0.52) | 0.17 (0.03/0.56) | 0.18 (-0.24/0.73) | 0.33 (0.09/0.75) | 0.26 (0.07/0.68) |
| kidneys | 0.96 (0.79/0.99) | 0.99 (0.96/1.00) | 0.98 (0.95/1.00) | 1.00 (1.00/1.00) | 1.00 (1.00/1.00) | 1.00 (1.00/1.00) |
| urinary bladder bottom | 0.11 (-0.07/0.56) | 0.63 (0.35/0.90) | 0.43 (0.19/0.80) | 0.17 (-0.14/0.69) | 0.76 (0.52/0.94) | 0.56 (0.30/0.87) |
| urinary bladder max fill | 0.18 (-0.02/0.62) | 0.38 (0.12/0.78) | 0.32 (0.11/0.72) | 0.43 (-0.05/0.85) | 1.00 (1.00/1.00) | 0.80 (0.59/0.95) |
| tumor | 0.74 (0.23/0.95) | 0.55 (0.23/0.87) | 0.61 (0.32/0.89) | 0.49 (0.01/0.87) | 0.99 (0.95/1.00) | 0.82 (0.62/0.96) |


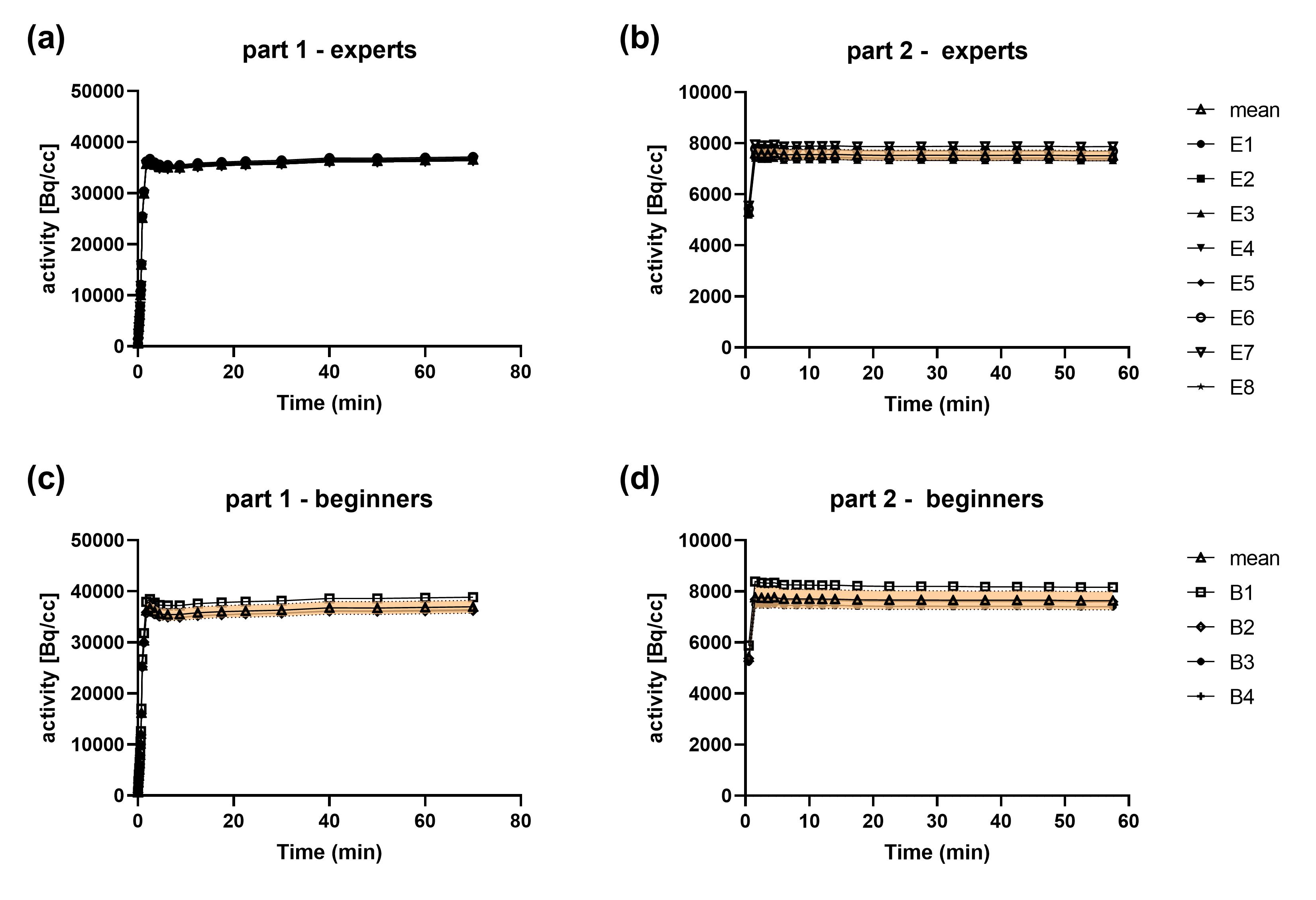


**Fig. s1** Whole field-of-view time-activity curves (TACs) obtained from all observers. The individual TACs obtained from the expert group (n=8) for (**a**) part 1 and (**b**) part 2 are shown. The TACs from the beginner group (n=4) for (**c**) part 1 and (**d**) part 2 of the study are illustrated. The mean value for each time point is overlaid (open triangle) and the standard deviation is illustrated as a filled orange area.

| 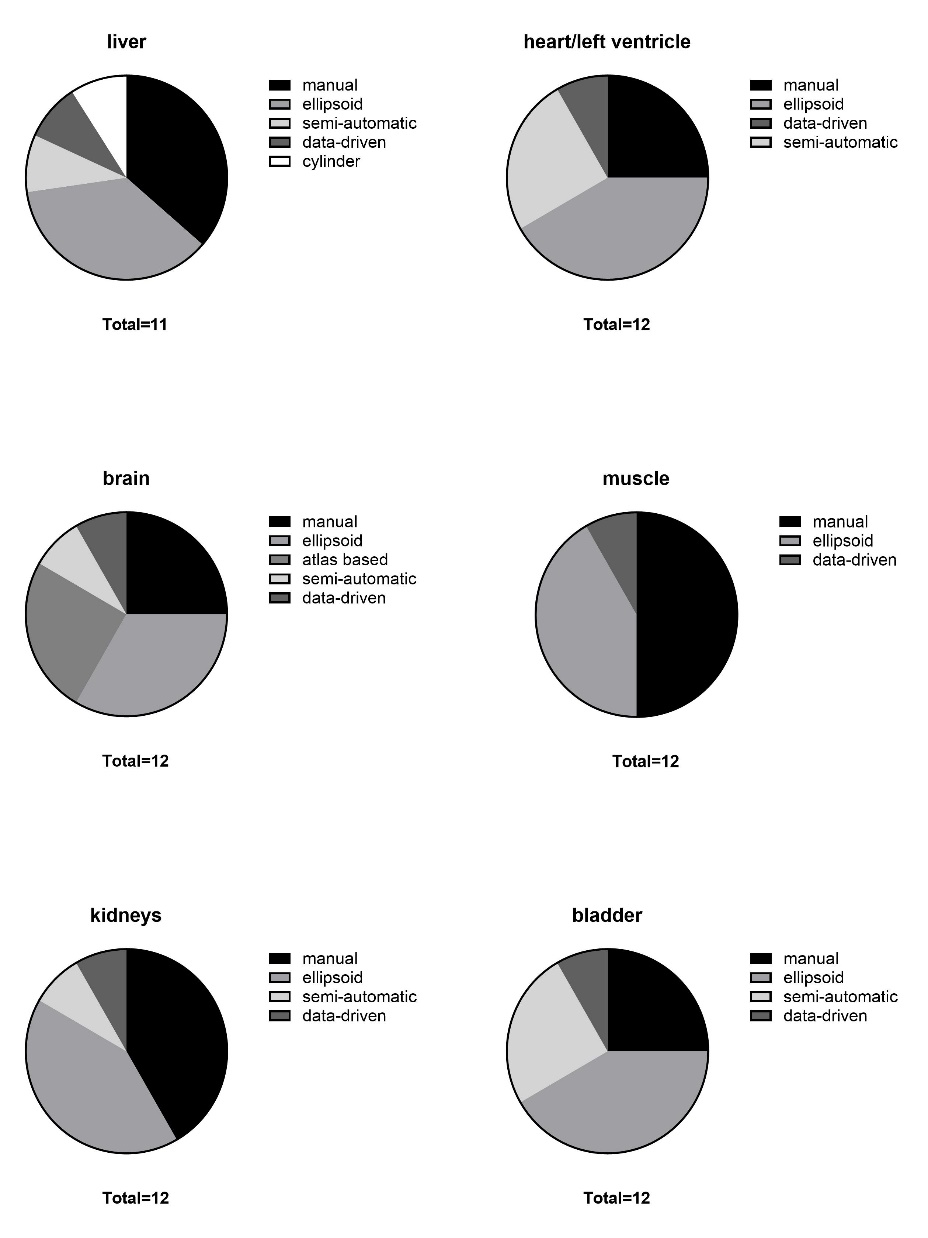 | **Fig. s2** VOI delineation methods applied by the observers when analyzing the dynamic [^18^F]FDG-PET-only datasets (part 1). (manual – manual delineation; ellipsoid, cylinder – use of fixed geometric objects; semiautomatic – use of thresholds; data-driven – segmentation based on tracer dynamics; atlas based – use of a defined brain atlas) (Abbreviations used: bladder – urinary bladder). |
| --- | --- |


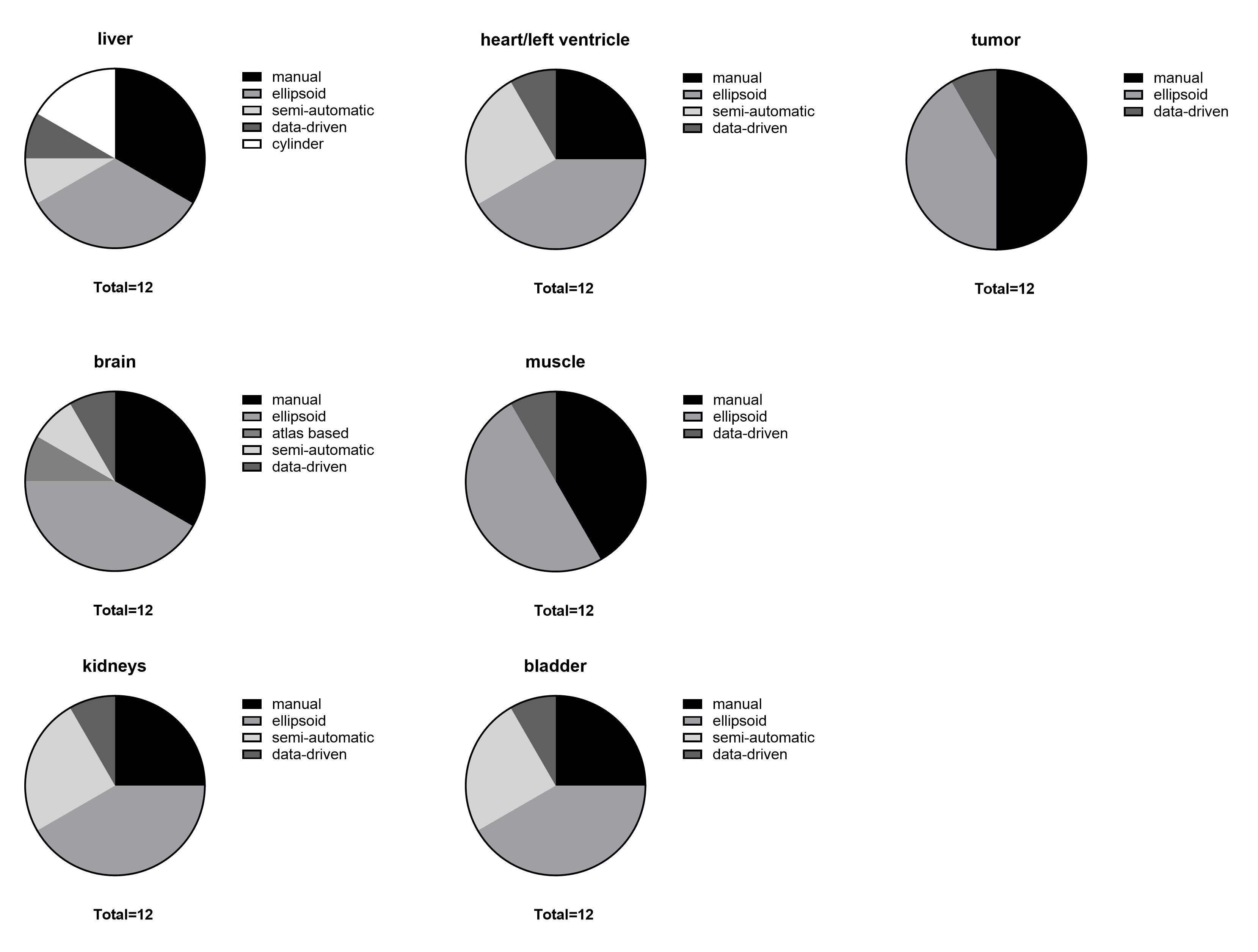


**Fig. s3** VOI delineation methods applied by the observers when analyzing the dynamic [^18^F]FDG-PET/CT datasets (part 2). (manual – manual delineation; ellipsoid, cylinder – use of fixed geometric objects; semiautomatic – use of thresholds; data-driven – segmentation based on tracer dynamics; atlas based – use of a defined brain atlas). (Abbreviations used: bladder – urinary bladder).


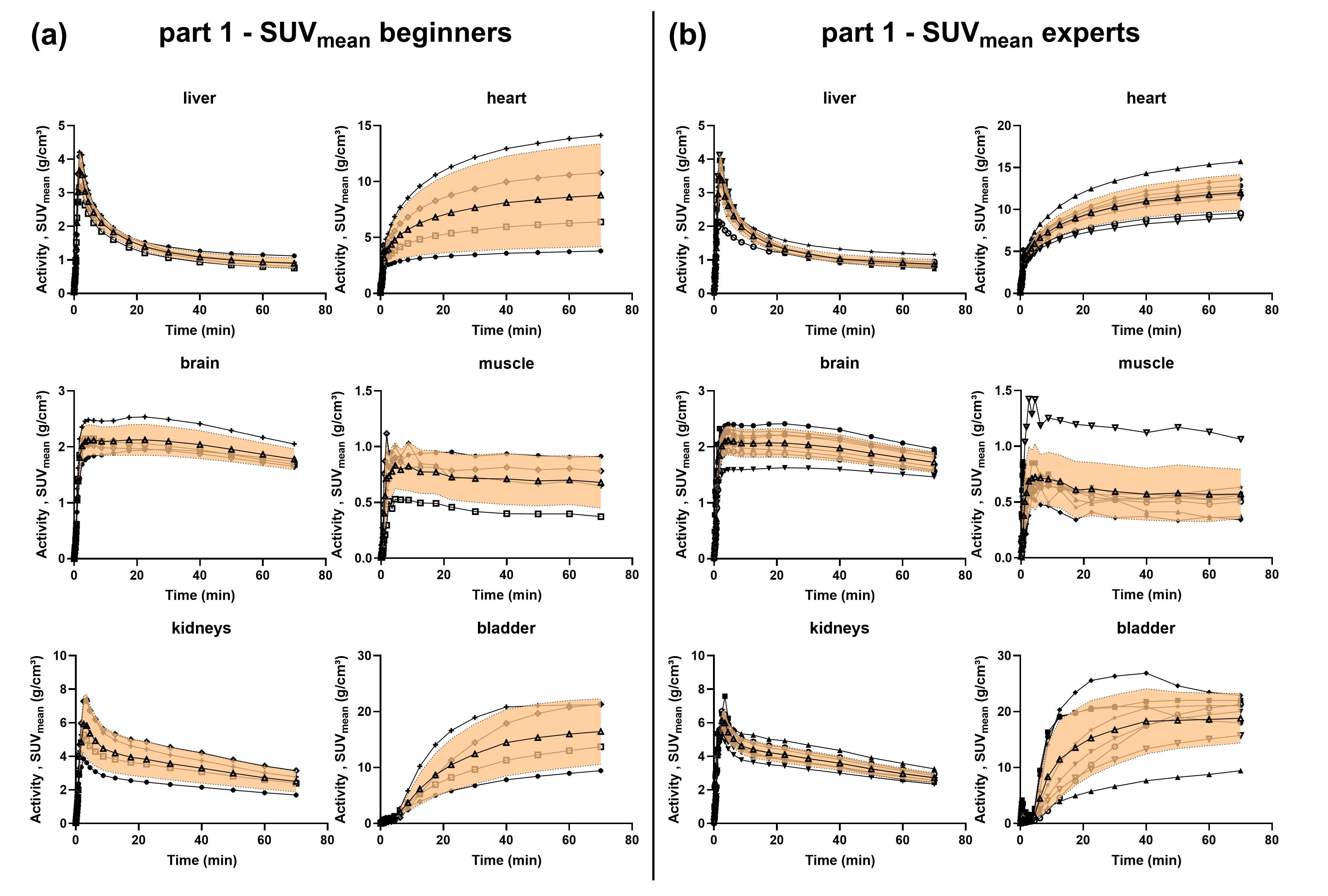


**Fig. s4** SUV_mean_ organ time-activity curves (TACs) extracted from one exemplary tumor-bearing mouse undergoing a dynamic [^18^F]FDG-PET scan. The individual TACs obtained from the (**a**) beginner group (n=4) and (**b**) expert group (n=8) are shown. The mean value for each time point is overlaid (open triangle) and the standard deviation is illustrated as a filled orange area. The kidney plot represents the mean value from the left and right kidneys, whereas the heart plot represents the mean value of the heart and left ventricle region. (Abbreviations used: bladder – urinary bladder).


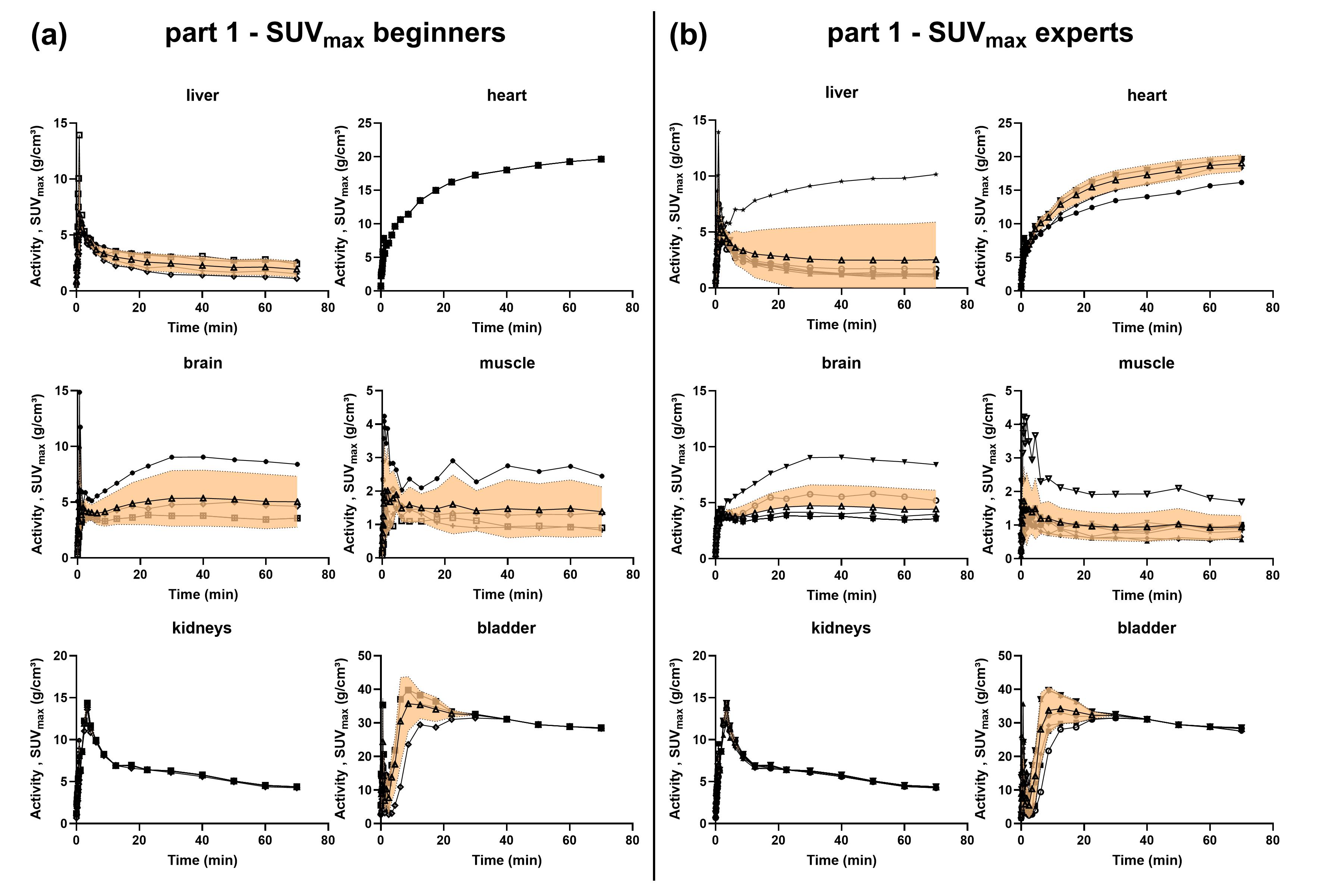


**Fig. s5** SUV_max_ organ time-activity curves (TACs) extracted from one exemplary tumor-bearing mouse undergoing a dynamic [^18^F]FDG-PET scan. The individual TACs obtained from the (**a**) beginner group (n=4) and (**b**) expert group (n=8) are shown. The mean value for each time point is overlaid (open triangle) and the standard deviation is illustrated as a filled orange area. The kidney plot represents the mean value from the left and right kidneys, whereas the heart plot represents the mean value of the heart and left ventricle region. (Abbreviations used: bladder – urinary bladder).


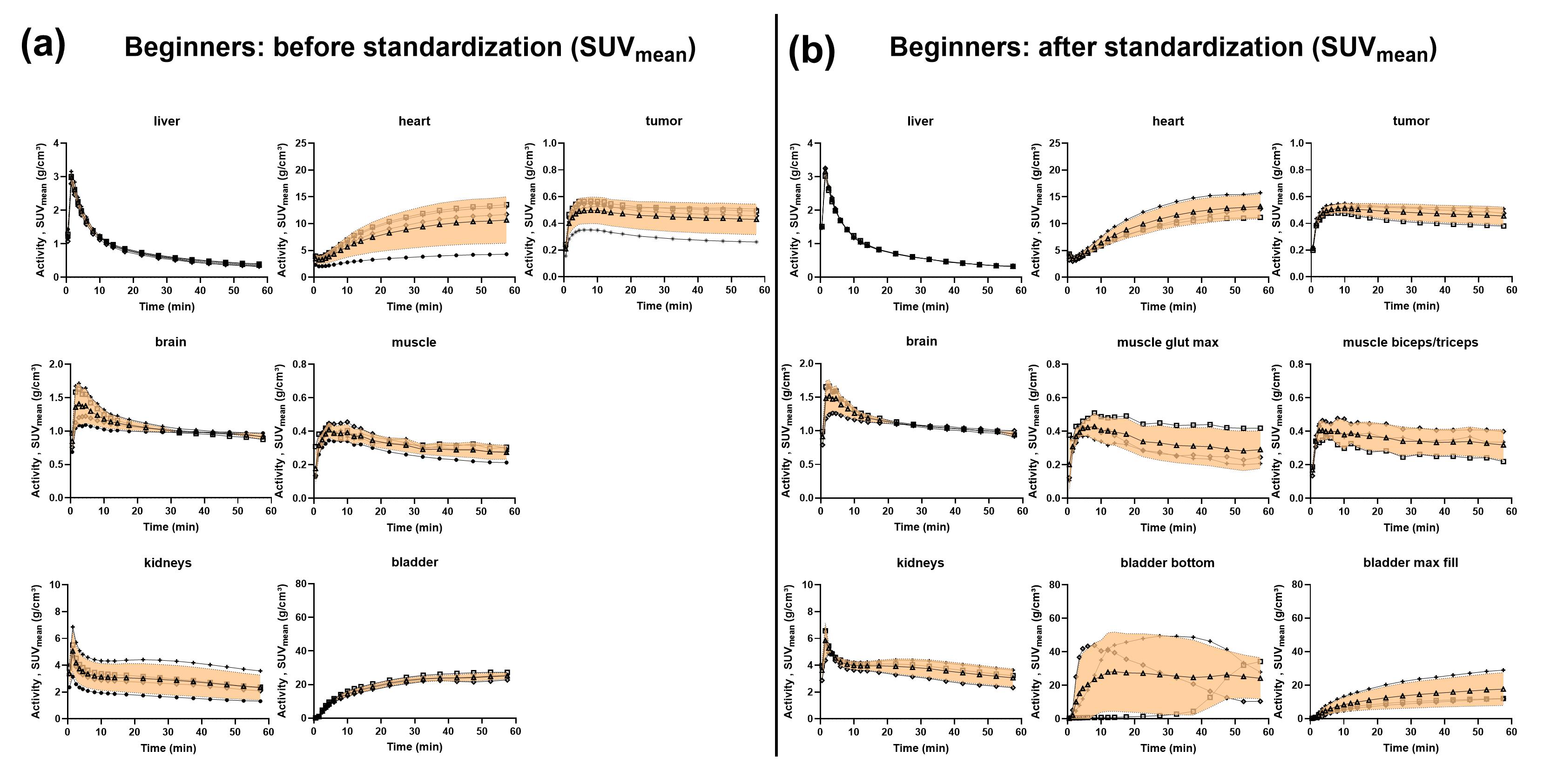


**Fig. s6** SUV_mean_ organ time-activity curves (TACs) extracted from one exemplary tumor-bearing mouse undergoing a dynamic [^18^F]FDG-PET/CT scan. The individual TACs obtained from the beginner group (n=4/3) (**a**) before (part 2) and (**b**) after standardization (part 3) are shown. The mean value for each time point is overlaid (open triangle) and the standard deviation is illustrated as a filled orange area. The kidney plot represents the mean value from the left and right kidneys, whereas the heart plot represents the mean value of the heart and left ventricle region. (Abbreviations used: bladder – urinary bladder, muscle glut max – muscle gluteus maximus, bladder bottom – bottom of the urinary bladder, bladder max fill – urinary bladder at maximum fill).


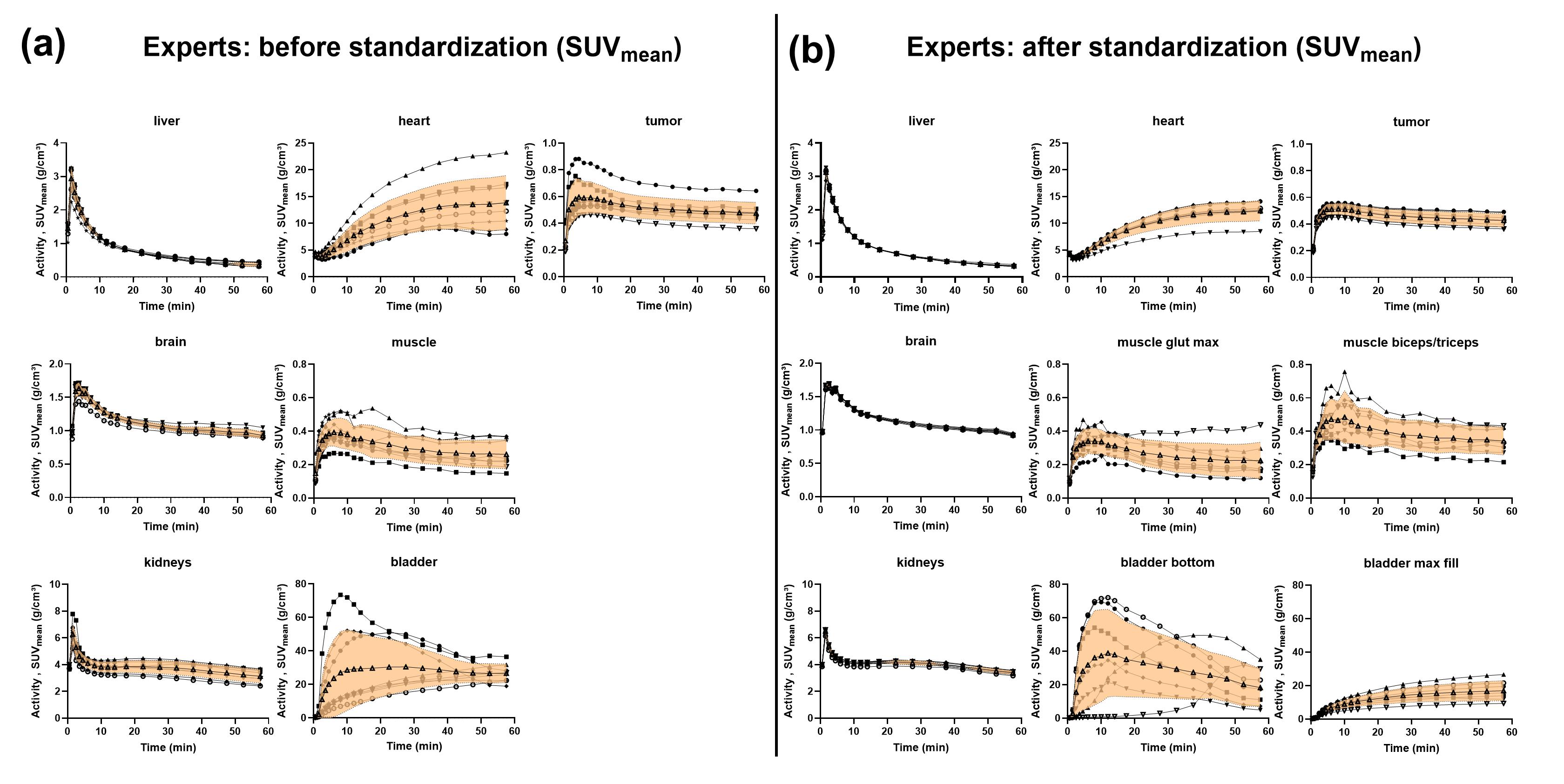


**Fig. s7** SUV_mean_ organ time-activity curves (TACs) extracted from one exemplary tumor-bearing mouse undergoing a dynamic [^18^F]FDG-PET/CT scan. The individual TACs obtained from the expert group (n=8/7) (**a**) before (part 2) and (**b**) after standardization (part 3) are shown. The mean value for each time point is overlaid (open triangle) and the standard deviation is illustrated as a filled orange area. The kidney plot represents the mean value from the left and right kidneys, whereas the heart plot represents the mean value of the heart and left ventricle region. (Abbreviations used: bladder – urinary bladder, muscle glut max – muscle gluteus maximus, bladder bottom – bottom of the urinary bladder, bladder max fill – urinary bladder at maximum fill).


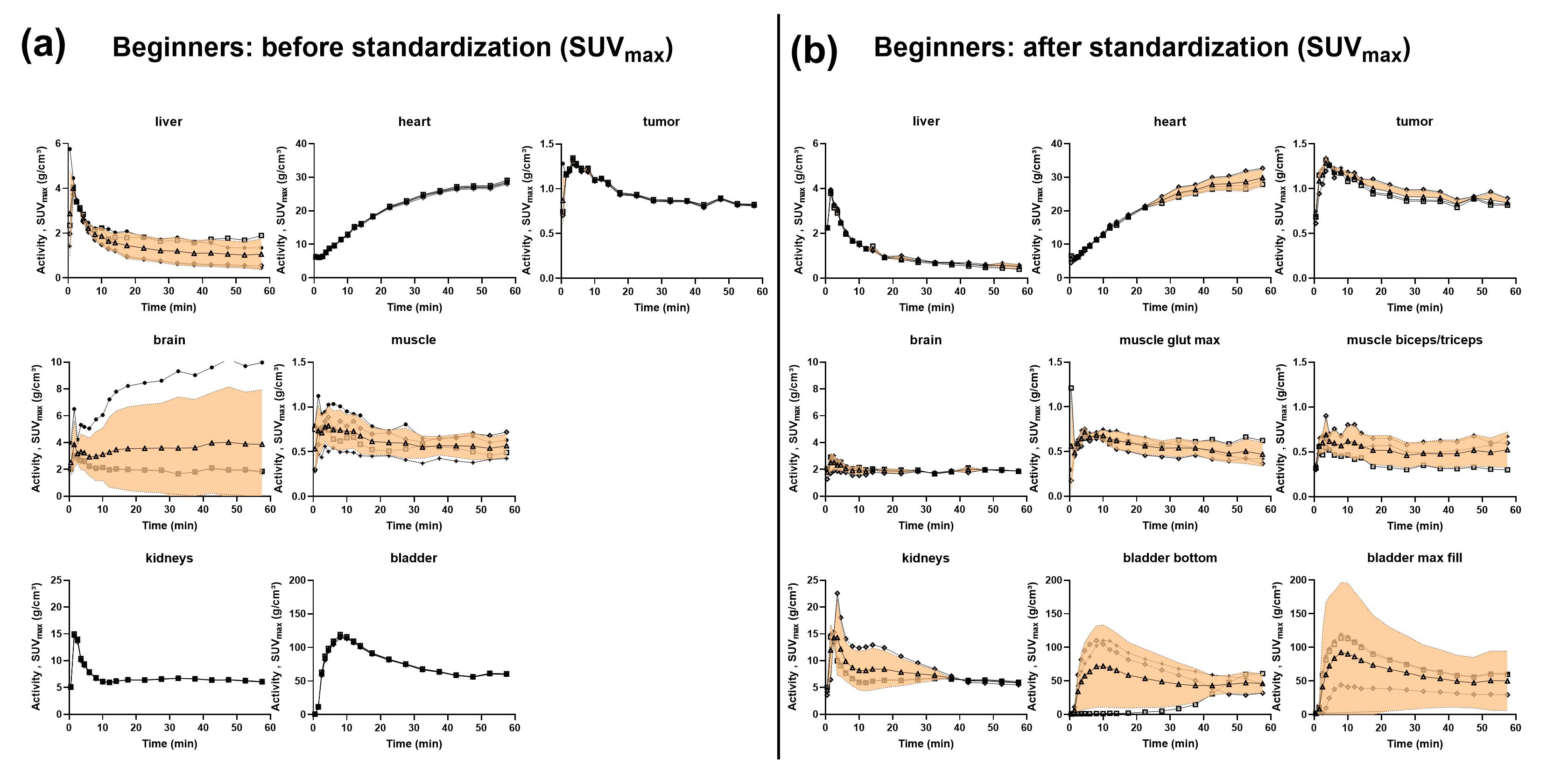


**Fig. s8** SUV_max_ organ time-activity curves (TACs) extracted from one exemplary tumor-bearing mouse undergoing a dynamic [^18^F]FDG-PET/CT scan. The individual TACs obtained from the beginner group (n=4/3) (**a**) before (part 2) and (**b**) after standardization (part 3) are shown. The mean value for each time point is overlaid (open triangle) and the standard deviation is illustrated as a filled orange area. The kidney plot represents the mean value from the left and right kidneys, whereas the heart plot represents the mean value of the heart and left ventricle region. (Abbreviations used: bladder – urinary bladder, muscle glut max – muscle gluteus maximus, bladder bottom – bottom of the urinary bladder, bladder max fill – urinary bladder at maximum fill).


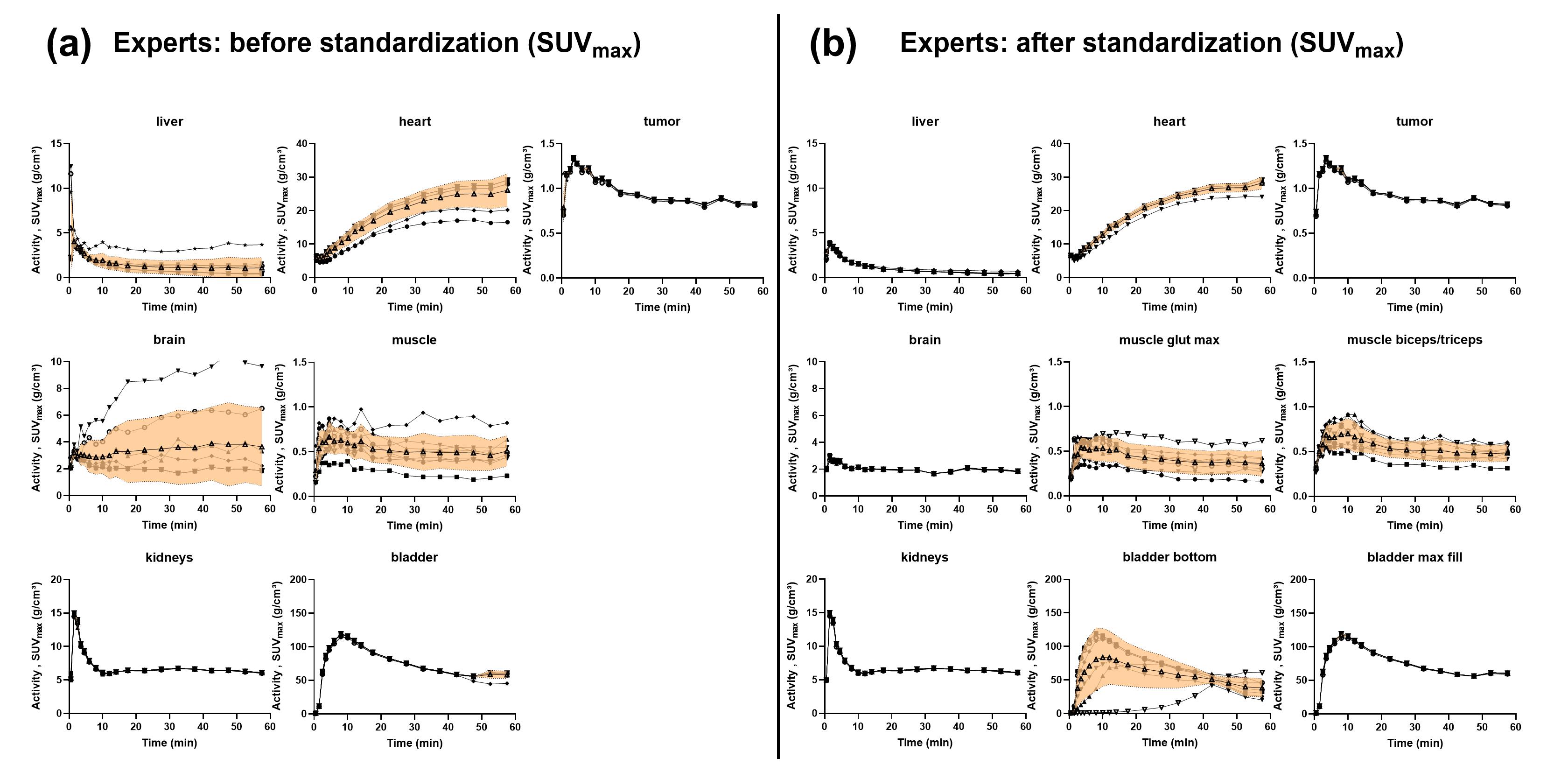


**Fig. s9** SUV_max_ organ time-activity curves (TACs) extracted from one exemplary tumor-bearing mouse undergoing a dynamic [^18^F]FDG-PET/CT scan. The individual TACs obtained from the expert group (n=8/7) (**a**) before (part 2) and (**b**) after standardization (part 3) are shown. The mean value for each time point is overlaid (open triangle) and the standard deviation is illustrated as a filled orange area. The kidney plot represents the mean value from the left and right kidneys, whereas the heart plot represents the mean value of the heart and left ventricle region. (Abbreviations used: bladder – urinary bladder, muscle glut max – muscle gluteus maximus, bladder bottom – bottom of the urinary bladder, bladder max fill – urinary bladder at maximum fill).


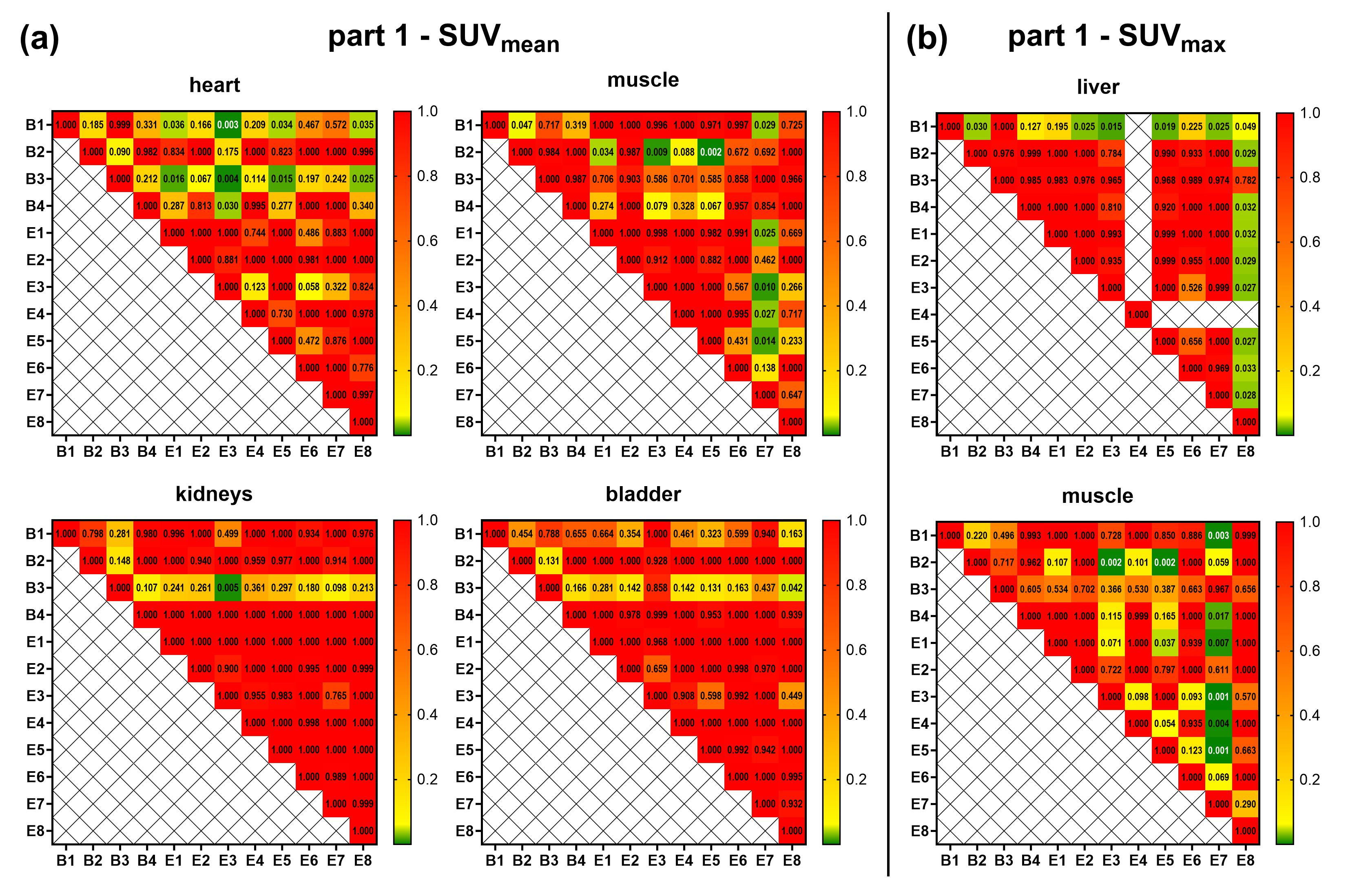


**Fig. s10** Heat maps derived from the (**a**) SUV_mean_ and (**b**) SUV_max_ analysis as a function of beginner or expert observers for [^18^F]FDG-PET-only data. B1-4: beginners 1 to 4; E1-8: experts 1 to 8. The displayed colors and numbers show the p values resulting from the Brown-Forsythe and Welch ANOVA followed by Dunnett’s T3 multiple comparisons test between individual observers. Only organs with significant comparisons (p<0.05) are shown. Expert 4 did not analyze the liver. (Abbreviations used: bladder – urinary bladder).


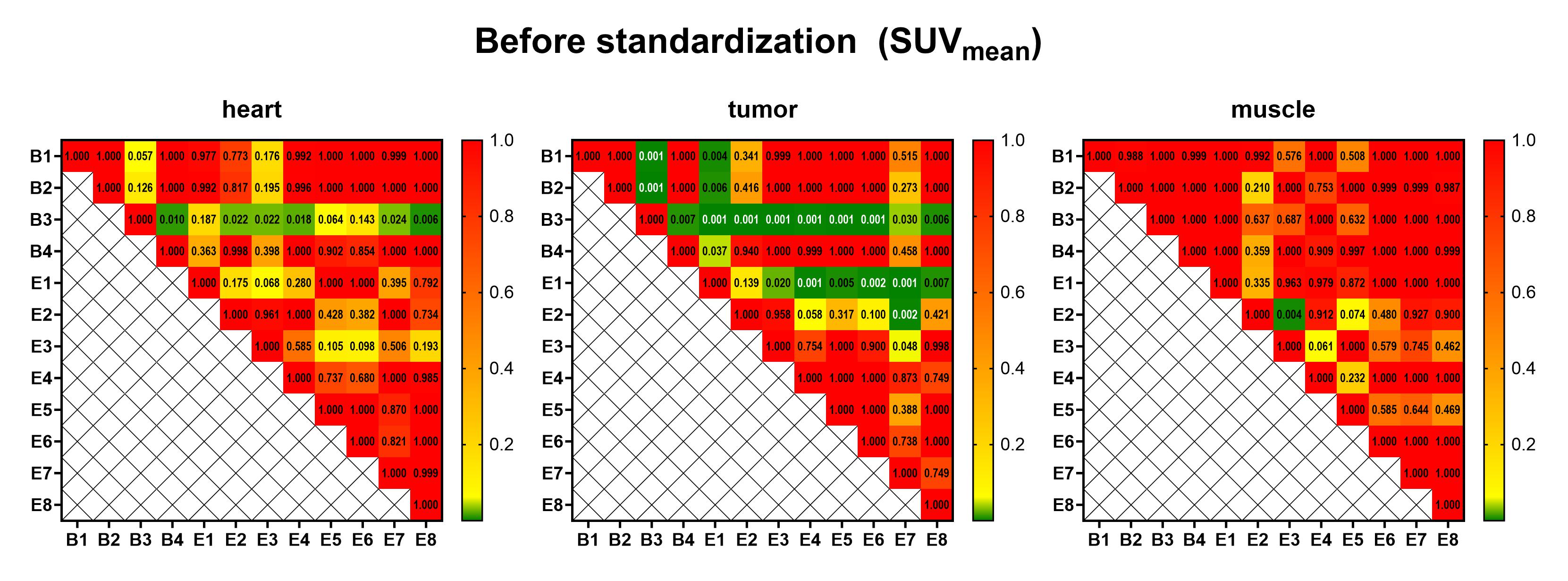


**Fig. s11** Heat maps derived from the SUV_mean_ analysis before standardization as a function of beginner or expert observers for [^18^F]FDG-PET/CT data. B1-4: beginners 1 to 4; E1-8: experts 1 to 8. The displayed colors and numbers show the p values resulting from the Brown-Forsythe and Welch ANOVA followed by Dunnett’s T3 multiple comparisons test between individual observers. Only organs with significant comparisons (p<0.05) are shown.


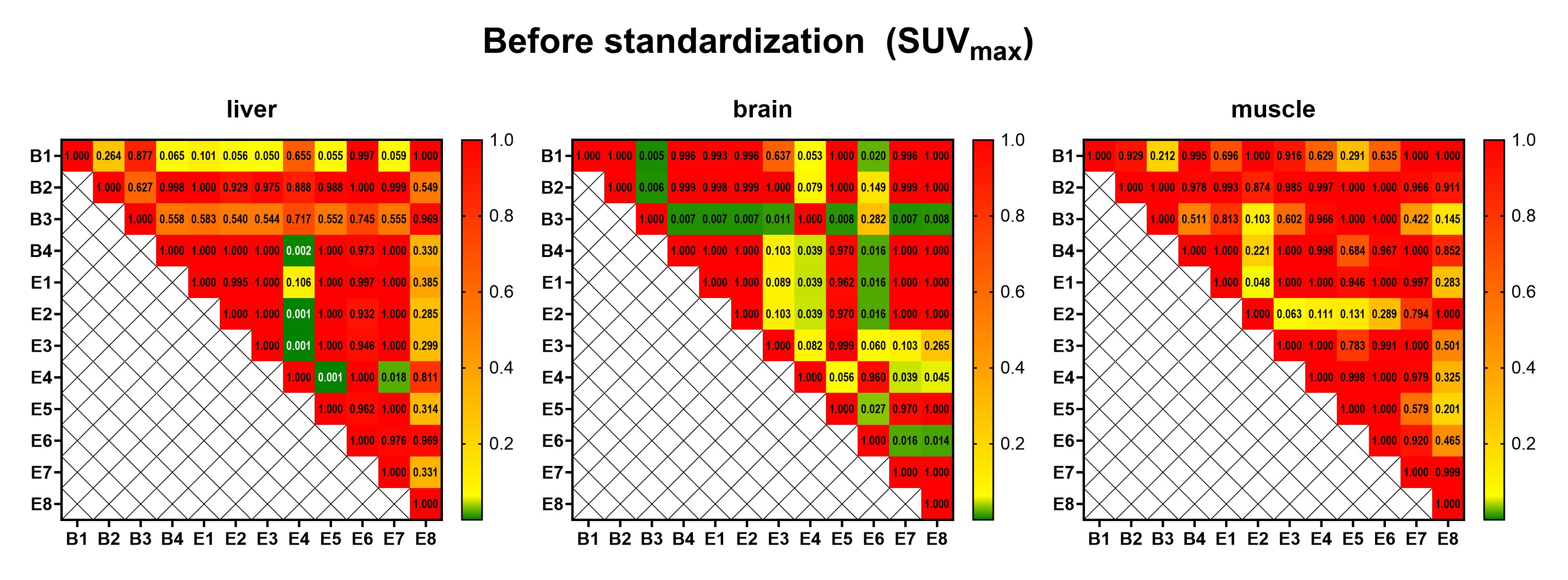


**Fig. s12** Heat maps derived from the SUV_max_ analysis before standardization as a function of beginner or expert observers for [^18^F]FDG-PET/CT data. B1-4: beginners 1 to 4; E1-8: experts 1 to 8. The displayed colors and numbers show the p values resulting from the Brown-Forsythe and Welch ANOVA followed by Dunnett’s T3 multiple comparisons test between individual observers. Only organs with significant comparisons (p<0.05) are shown.

| 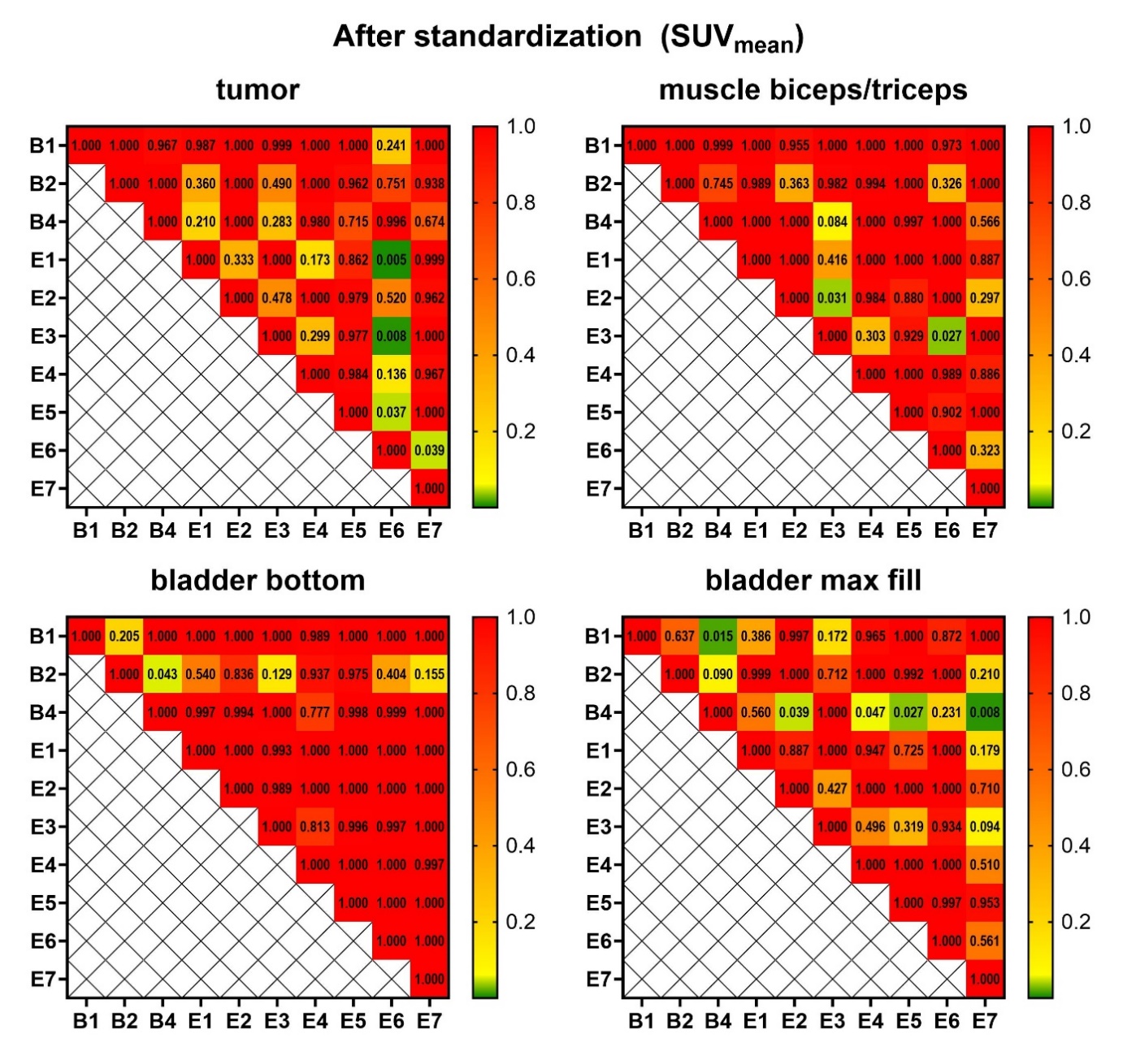 | **Fig. s13** Heat maps derived from the SUV_mean_ analysis after standardization as a function of beginner or expert observers for [^18^F]FDG-PET/CT data. B1-4: beginners 1 to 4; E1-8: experts 1 to 8. The displayed colors and numbers show the p values resulting from the Brown-Forsythe and Welch ANOVA followed by Dunnett’s T3 multiple comparisons test between individual observers. Only organs with significant comparisons (p<0.05) are shown. (Abbreviations: bladder bottom – bottom of the urinary bladder, bladder max fill – urinary bladder at maximum fill). |
| --- | --- |
